# Women in the triathlon—the differences between female and male triathletes: a narrative review

**DOI:** 10.3389/fspor.2025.1567676

**Published:** 2025-06-06

**Authors:** Michèle Loosli, Pantelis T. Nikolaidis, Volker Scheer, Matthias Wilhelm, Pedro Forte, Marilia Andrade, Thomas Rosemann, Sasa Duric, Ivan Cuk, Beat Knechtle

**Affiliations:** ^1^ Faculty of Medicine, University of Bern, Bern, Switzerland; ^2^ School of Health and Caring Sciences, University of West Attica, Athens, Greece; ^3^ Ultra Sports Science Foundation, Pierre-Benite, France; ^4^ Centre for Rehabilitation & Sports Medicine, Bern University Hospital, University of Bern, Bern, Switzerland; ^5^ Department of Sports, Higher Institute of Educational Sciences of the Douro, Penafiel, Portugal; ^6^ CI-ISCE, ISCE (Instituto Superior de Ciências Educativas) Douro, Penafiel, Portugal; ^7^ Department of Sports, Instituto Politécnico de Bragança, Bragança, Portugal; ^8^ Research Center for Active Living and Wellbeing (LiveWell), Instituto Politécnico de Bragança, Bragança, Portugal; ^9^ Federal University of São Paulo, São Paulo, Brazil; ^10^ Institute of Primary Care, University of Zurich, Zurich, Switzerland; ^11^ Liberal Arts Department, American University of the Middle East, Egaila, Kuwait; ^12^ Faculty of Sport and Physical Education, University of Belgrade, Belgrade, Serbia; ^13^ Medbase St. Gallen am Vadianplatz, St. Gallen, Switzerland

**Keywords:** ironman, sex differences, performance, endurance, ultra triathlon, training, female triathletes

## Abstract

**Introduction:**

Triathlon events have gained popularity in recent years. With the increasing participation of women, aspects that influence performance and physiology, as well as differences between women and men, are of interest to athletes and coaches. A review of the existing literature concerning differences between women and men in triathlon is lacking. Therefore, this narrative review aimed to compare female and male triathletes in terms of participation, performance, and the different influences on performance (e.g., physiology, age, pacing, motivation).

**Methods:**

A literature search was conducted in PubMed and Scopus using the search terms “female triathletes”, “women in triathlon”, “triathlon AND gender difference”, and “triathlon AND sex difference”. 662 articles were found using this search strategy, of which 147 were relevant for this review. All distances from sprint to ultra-triathlon (e.g., x-times IRONMAN® distance) were analyzed.

**Results:**

The results showed that the participation of female triathletes, especially female master triathletes increased over time. An improvement in the performance of female and older triathletes was observed at the different distances in the last decades. Sex differences in performance varied across distances and in the three disciplines. Female triathletes showed a significantly lower VO2max and higher lactate thresholds compared to men. They also had a higher body fat percentage and lower body mass. The age for peak performance in the IRONMAN® triathlons is achieved between 25 and 39 years for both women and men. Strong predictors of IRONMAN® race performance in both female and male triathletes include achieving a personal best time in a marathon and a previous best time in triathlon races.

**Conclusion:**

Further studies need to balance the representation of female and male athletes in study cohorts to ensure that findings are relevant to both sexes. Another research gap that should be addressed by future studies is the effect of menstruation and female hormones, the presence of premenstrual syndrome, and the impact of pregnancy and childbirth on the triathlon performance to better understand the differences with men and to account for hormonal fluctuations in training.

## 1 Introduction

Triathlon is a unique endurance sports discipline in which swimming, cycling and running are combined and performed one after the other (1). The length of the individual disciplines varies depending on the length of the event. The different distances are shown in Table 1 (2, 3). Distances longer than the IRONMAN® distance are typically referred to as ultra-triathlons (3). Each of these distances places specific demands on the athletes’ physical performance, physiological systems, training preparation and recovery strategies.

**Table 1:** The different triathlon lengths in kilometres.

Distance	Swimming	Cycling	Running
Sprint	0.75	20	5
Olympic	1.5	40	10
IRONMAN® 70.3	1.9	90	21.1
IRONMAN®	3.8	180	42.2
Double Iron ultra-triathlon	7.6	360	84.4
Triple Iron ultra-triathlon	11.4	540	126.6
Deca Iron ultra-triathlon	38	1,800	422

In recent years, endurance events such as marathons and triathlons have become increasingly popular (4–7). In triathlon events, the IRONMAN® triathlon is especially popular, as both the number of races and athletes increase annually (8). Due to the increasing interest in participation in triathlon, numerous studies have been published in recent years addressing various aspects of such races, including participation trends (3, 9–11), performance trends over the years (12–14), predictive variables of performance (15–18), the different characteristics of participants (19–22), age-related aspects (23–27), training (28–30), nutrition (31, 32) and pacing (33–36).

With increasing participation, the performance also changed. In the Ironman World Championship “IRONMAN® Hawaii”, both elite (24) and age group athletes (37) have improved their performance over the last decades. In contrast, Sousa et al. showed a negative trend in the performance of women and men in ultra-triathlon (Double Iron, Triple Iron, Quintuple Iron (19 km swimming, 900 km cycling, and 211 km running) and Deca Iron ultra-triathlon between 1985 and 2018 (38). Several studies have aimed to identify the factors influencing endurance performance in running and triathlon. Success in endurance events is determined by a complex interplay among various factors, including oxidative capacity, the energy cost of locomotion, substrate efficiency, fatigue resistance and musculoskeletal conditioning, race nutrition, gastrointestinal function, age, sex, experience, pain management, decision-making, and motivation and psychological disposition (14, 34, 39–44).

While triathlon was originally heavily dominated by men, the number of female participants has increased significantly in recent decades. Women have made significant progress in both amateur and professional sports and have increasingly established themselves in competitions (45–47). Nevertheless, there are still striking differences in participation rates, performance, and physiological requirements between female and male athletes.

Since the number of female triathletes has increased and sex influences on performance, significant attention has been paid to the sex difference in endurance sports performance, notwithstanding the fact that the number of scientific studies involving the female population remains significantly lower than those involving the male population (48). Triathlon provides an intriguing alternative model to analyze the sex difference in endurance performance because the sex difference can be analyzed both for the same subject as a whole and for the three disciplines separately (3, 13, 25, 49). With the increase in women's participation in amateur and elite endurance sports over the past three decades, the sex performance gap appears to be narrowing (7, 10). This also appears to be associated with improvements in women's performance in recent decades (45–47). Some authors have wondered whether the sex gap in endurance performance will close (50–52). However, more recent studies have shown that the sex gap is no longer narrowing (53, 54).

The gap between sexes in endurance performance is a crucial subject for athletes, coaches and researchers in sports science and sports medicine. A comprehensive understanding of these differences is important for identifying the challenges and opportunities specific to women in triathlon and for developing strategies to promote sex equality and participation in sports. According to a recent consensus of the American College of Sports Medicine, women had a 10%–30% lower performance in sports, where endurance and muscle power played a dominant role (55). The aim of the present narrative review is therefore to provide insights into the differences between female and male triathletes in terms of participation, performance and the various influences on performance (e.g., physiology, age, pacing, motivation). The main focus is on the extent to which the performance of women and men differs at different triathlon lengths and which physiological and psychological factors influence these differences. In addition to the obvious differences in performance, the numerous social, cultural and biological factors that can play a role are also taken into account.

## 2 Methods

To comprehensively examine the participation and performance trends of women in triathlon as well as the differences between women and men, a narrative review was conducted to identify, select and analyze relevant literature. For this narrative review, a literature search was performed using PubMed and Scopus databases. The search was not limited by publication date; all studies published until November 2024 were considered.

The following terms were used for the literature search: “female triathlete”, “women in triathlon”, “triathlon AND gender difference”, “triathlon AND sex difference”. All studies on women's participation, performance and influencing factors in triathlon (e.g., physiology, age, training, motivation, pacing, experience) were included. Studies that did not specifically address these topics or focused exclusively on male athletes were excluded.

**Figure 1:**
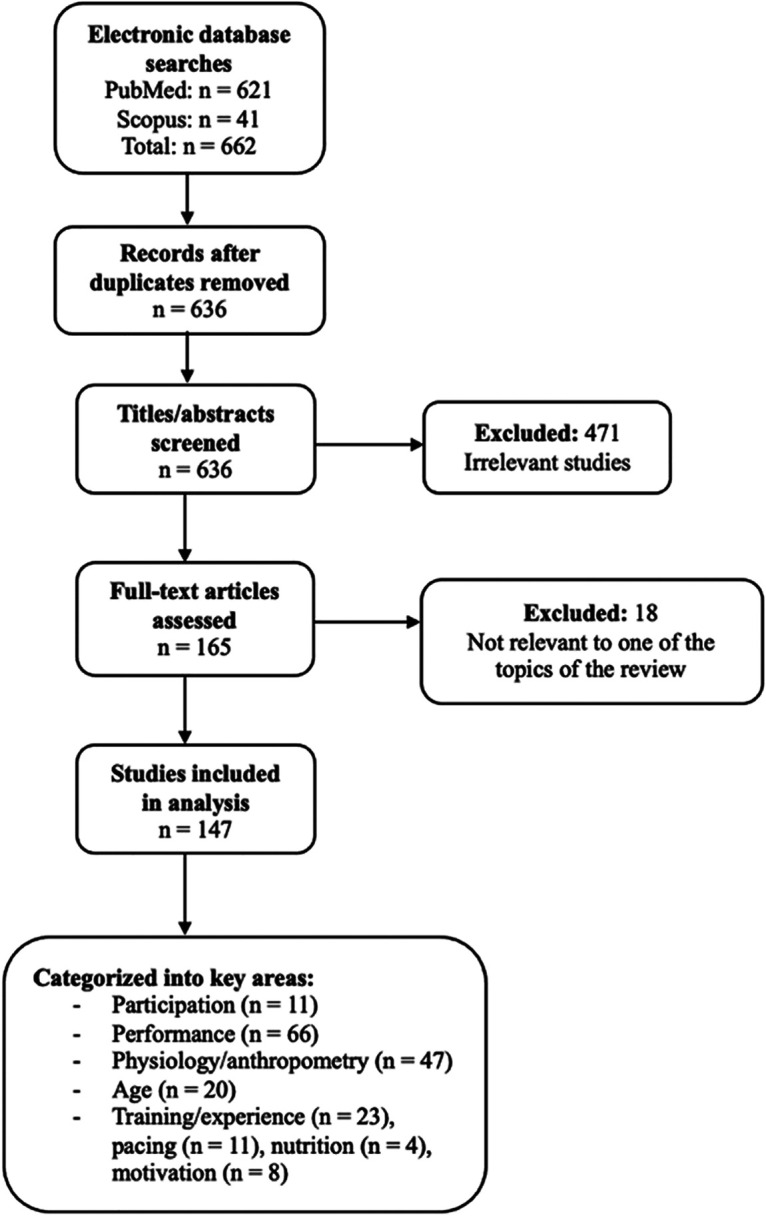
Flowchart of the searching strategy.

Figure 1 shows the flowchart of the searching strategy used.

Following the literature search, the identified studies were categorized based on their research focus according to the main topics of interest of the present narrative review. The findings of the literature search were systematically recorded in tables. These tables contain the key information for each article, including the number of participants, the triathlon distance, the observation of the study and the main findings. This ensured that the overview of the relevant literature was clearly structured. Several studies were included twice or more in each related category of interest because they focused on two or more of the areas of interest.

## 3 Results and discussion

The focus of the present narrative review was on participation (11 studies) and performance trends (66 studies) of women at different triathlon distances and the factors influencing performance, such as aspects of physiology and anthropometry (47 studies), age (20 studies), training/experience (23 studies), pacing (11 studies), nutrition (4 studies) and motivation (8 studies).

### 3.1 Participation

A total of 11 studies focused on the participation trends of female triathletes were identified. Participation in the IRONMAN® 70.3 (6), the IRONMAN® (7) and the ultra-triathlon (e.g., Double and Triple Iron) (9, 56) has steadily increased for both sexes, while numbers in Quadruple to Deca Iron events remain stable (3, 57). No major changes in participation were also observed in the Olympic distance triathlon (58). This is also supported by the analysis of the Olympic distance “Zurich Triathlon” between 2000 and 2010, where only the participation of female triathletes aged 40–54 years increased (47). However, no studies could be found with current numbers of participants at the Olympic distance.

**Figure 2:**
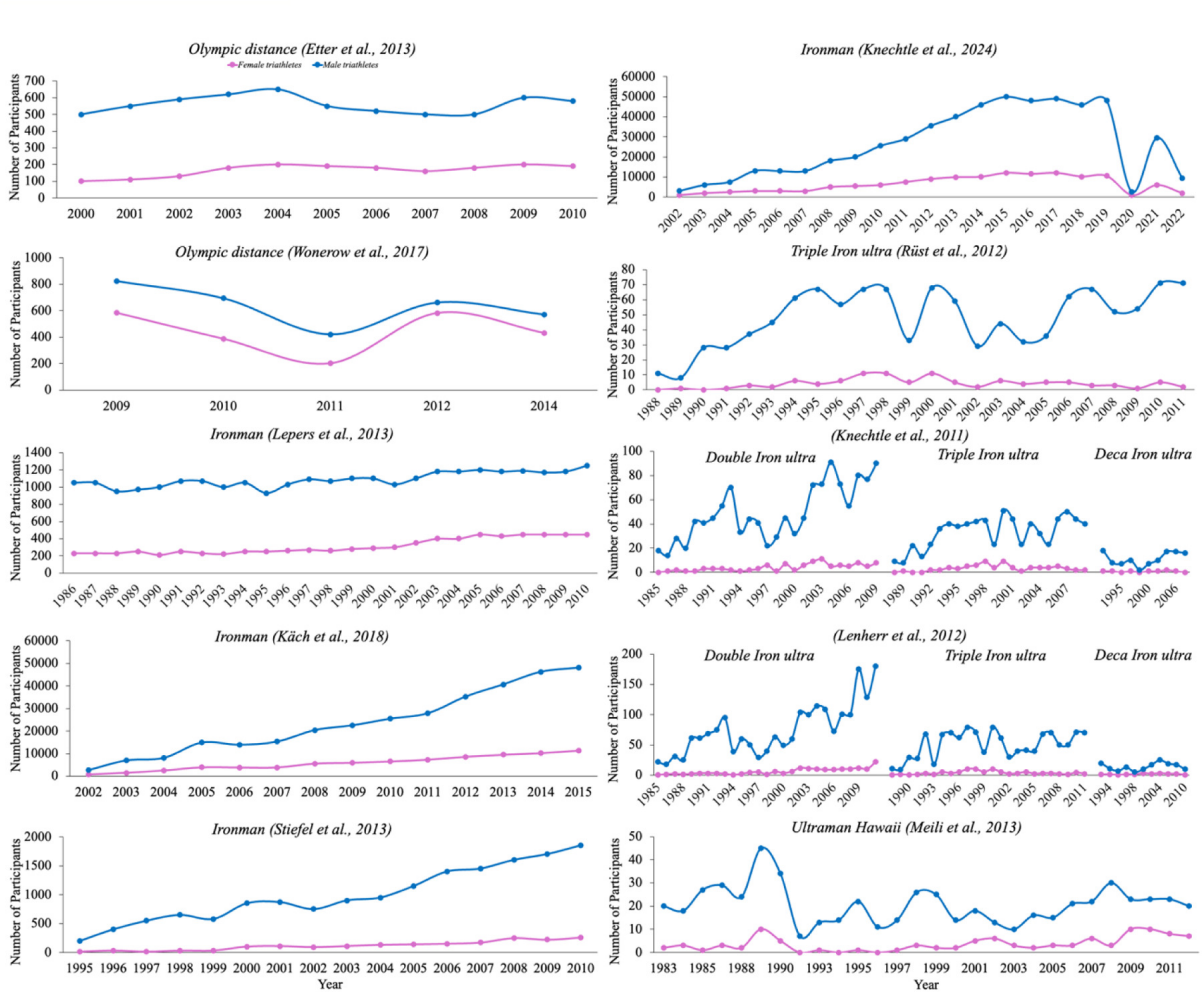
The participation rate trends across different studies (3, 9, 11, 37, 47, 57, 58, 60, 225, 226).

Figure 2 illustrates participation trends in female and male triathletes across different time periods and race distances, as reported by various studies. The figure highlights differences in participation rates, showing that despite an increase in female participation over time, the number of male triathletes remains consistently higher.

Table 2 shows the main findings of studies investigating the changes in participation in different triathlon distances over time.

**Table 2:** Overview of the key findings of studies investigating the participation trends in different triathlon distances.

Subjects	Distance	Observation	Relevant findings	Reference
*N* = 7,939, female = 1,666	Olympic distance	Participation at the Olympic distance “Zürich Triathlon” (1.5 km swim, 40 km cycle and 10 km run) from 2000 to 2010	• Female triathletes aged from 40 to 54 years increased their participation, while the participation of younger women and men remained stable. • Females accounted for 26.4 ± 5.8% of the field since 2000. • The age group with the largest participation has been from 35 to 39 years for men and from 30 to 34 years for women.	(47)
*N* = 6,484, female = non specified	Olympic distance	Participation trends of age group athletes at the ITU World Championships over the Olympic distance triathlon from 2009 to 2014	• No significant change in the number of female or male participants for any of the age groups was demonstrated from 2009 to 2014.	(58)
*N* = 6,303, female = 1,115	IRONMAN® 70.3 distance	Participation rate in the IRONMAN® 70.3 Switzerland from 2007 to 2010	• Women accounted for an average of 18.0%±1.9% of the triathletes since 2007. • The greatest participation rates were 30–34 years for women and 35–39 years for men.	(227)
*N* = 35,293, female = 7,825	IRONMAN® distance	Changes in participation of masters triathletes at the IRONMAN® triathlon World Championship from 1986 to 2010	• The participation of masters triathletes increased during the 1986–2010 period. • Female triathletes accounted on average for 22 ± 3% (range 18%–28%) of the field over the 25-year period. • For both women and men, there was a significant rise in relative participation among age groups ≥40 years, while the relative participation significantly decreased among both women and men <40 years, especially for the youngest age groups 18–29 years.	(37)
*N* = 410,881, female = 81,815	IRONMAN® distance	Participation of all pro and age group triathletes ranked in all IRONMAN® triathlons held worldwide between 2002 and 2015	• The number of finishers increased significantly in all female age groups from 18 to 24 to 70–74, except age group 75–79 years.	(225)
*N* = 17,786, female = 1,847	IRONMAN® distance	Participation trends in IRONMAN® Switzerland from 1995 to 2010	• There was a progressive rise in the number of finishers since 1995 for both sexes. • Women accounted for 10–14% of the field since 2001.	(226)
*N* = 687,696, female = 134,088	IRONMAN® distance	Participation trends of official IRONMAN® races worldwide between 2002 and 2022	• The number of male finishers increased more than the number of female finishers. • The men-to-women ratio increased over the years.	(60)
*N* = 1,847, female = 503 (finished “IRONMAN® Hawaii”)	IRONMAN® distance	Comparison of participation of athletes in various age groups between “IRONMAN® Hawaii” and its qualifier races in 2010	• Female finishers were overrepresented and male finisher underrepresented in “IRONMAN® Hawaii” compared with in the Ironman qualifiers. • The percentage of women overall finishers was higher in “IRONMAN® Hawaii” (27.2%) than in the IRONMAN® qualifiers (18.9%). • In “IRONMAN® Hawaii”, the percentage of female finishes was lower for American and Canadian but higher for German athletes.	(10)
*N* = 1,256 (starters), female = 102	Triple Iron ultra-triathlon distance	Changes in participation in Triple Iron ultra-triathlon across years from 1988 to 2011	• During the studied period, the number of finishers were 824 (71.4%) for men and 80 (78.4%) for women. • Participation increased for men while it remained stable for women (8%).	(11)
*N* = 3,057, female = 257	Double—Deca Iron ultra-triathlon distance	Changes in participation trends in ultra-triathlon between 1985 and 2005	• The number of finishers increased in Double and Triple Iron, while it remained stable in Deca Iron. • Women accounted for −8%–10% of the starters. • With increasing length of the races, the number of participants dramatically decreased.	(3)
*N* = 3,579, female = 300	Double—Double Deca Iron ultra-triathlon distance	Participation trends in ultra-triathlon between 1985 and 2011	• The number of male participants in Double and Triple Iron ultra-triathlon increased, while the number only increased in Double Iron ultra-triathlon. • For the other distances (Quadruple to Double Deca Iron), no increased in participation was found.	(57)
*N* = 668, female = 98	“Ultraman® Hawaii” (day 1 with 10-km swimming and 145-km cycling, day 2 with 276-km cycling, and day 3 with 84-km running)	Changes in participation in “Ultraman® Hawaii” from 1983 to 2012	• The number of female finishers increased from 3 women (14% of the total field) in 1984 to 7 women (26%) in 2012, while the number of male finishers remained stable.	(9)

ITU, International Triathlon Union.

#### 3.1.1 Female participation

The number of female participants has progressively increased since 1980 (37). In the 1981 “IRONMAN® Hawaii” event, there were 20 female finishers (6% of participants), while in 2010 the number rose to 470 (27% of participants) (7, 37). A look at the IRONMAN® Triathlon World Championship between 1983 and 2012 shows that the number of female participants increased more rapidly than the overall number of participants. The number of male participants rose from 720 to 1362 during this period, which corresponds to a relative increase of 189%, while the number of women increased by 455% (from 115 to 524 participants) (59). This trend can also be observed in the marathon running. In a study between 1980 and 2009, Lepers et al. showed that the participation of female marathon runners increased at a higher rate (5). In contrast, a study investigating sex differences in IRONMAN® races worldwide between 2002 and 2022 found that the number of male finishers increased more than the number of female finishers, leading to an increase in the men-to-women ratio over the years (60).

The reasons for the increase in female participation are not clear. Still, social phenomena such as an enhanced focus on personal physical fitness and the positive impact of a healthy lifestyle on longevity could have been potential explanations (61). The increasing social acceptance of “active” women may also have played a role in the increased participation of women (62). Furthermore, greater media visibility of female athletes, targeted initiatives to promote women in endurance sports, and a broader societal shift toward sex equality in sport may have contributed to this trend. Role models in elite sports and the growing availability of women-only races or training groups could also be motivating factors.

In the context of age group triathletes, the rise in the number of female participants has surpassed that of male participants (7). Increased life expectancy and improved training opportunities for master athletes could explain the growing participation in endurance events in recent years (37).

Despite the increase in female participants, the proportion of women in triathlon events remains lower compared to men, with the female rate varying between 25% and 40% of the total field (13, 37, 58). In contemporary times, female triathletes have access to comparable training and competitive opportunities as their male counterparts across most regions globally. Nonetheless, female participation rates persistently lag below those of men, particularly in long-distance triathlon events (63). The female participation rate seems to have declined in the IRONMAN® to Double Deca Iron ultra-triathlon between 1978 and 2013. Rüst et al. found the highest percentage of women at the “IRONMAN® Hawaii” with 22.1% and the lowest at the Deca Iron ultra-triathlon with 6.5% (64). The analysis of the Triple Iron ultra-triathlon between 1988 and 2011 indicated that the number of female participants remained constant at 8%, while overall participation, especially among men, increased over this period (11). Other studies have also found a low participation rate of female triathletes in ultra-triathlons (3, 65). Additionally, due to the different men-to-women ratios in the different race lengths, the ratio also varied across the age groups. A study investigating sex differences in IRONMAN® age group triathletes found that the ratio increased with increasing age (60).

In contrast to other traditional endurance events such as marathons, the participation rate of female athletes in triathlons, particularly at the IRONMAN® distance, persists at a lower level (7). However, the rate is higher than for ultra-endurance events like a 161-km ultra-marathon (7). Knechtle et al. have recently shown that female runners participate more often in shorter race distances and less often in marathons and ultra-marathons, and that the male-to-female ratio increases with increasing race distance (66). The lower rate of women finishers in longer race distances is also consistent with the findings in duathlon (67). This might be explained by motivational reasons, differences in training practices and sociocultural contexts (7, 68, 69).

Data from Hunter and Stevens provided evidence that lower female participation in competitions such as marathon running and less depth among women competitors could exacerbate the sex difference in running speed beyond the sole physiological sex difference (70).

Future research on triathlon participation should place greater focus on current trends among female athletes, as recent data in this area remain limited. In addition, studies should consider the factors influencing female athletes’ engagement in the sport. Understanding the barriers to participation, such as the availability of resources, training support, and the possible impact of social and economic limitations based on sex, should receive careful consideration. Further investigation into the specific motivations driving female participation, and how these may differ from or align with those of male athletes would also provide valuable insights.

### 3.2 Performance

#### 3.2.1 Changes in performance across years

In addition to the increase in participation in recent decades, there has also been an increase in performance. Both elite and non-elite female and male triathletes have demonstrated performance improvements and sex differences in performance have received considerable attention over the last few decades (7, 37, 63, 64, 71).

Table 3 lists the key findings of studies that have examined changes in performance at different triathlon distances across years. In terms of overall race time, women have shown greater improvements compared to men. For example, during the period between 1995 and 2011 at the “IRONMAN® Switzerland”, the top 10 elite women demonstrated a more pronounced improvement in total time by 12.7%, whereas the top 10 elite men improved their total time by 6.4% (72). It should be noted that women accounted for 10.6% of all finishers in this study.

**Table 3:** Overview of key findings from studies investigating performance trends in different triathlon distances across the years.

Subjects	Distance	Observation	Outcome	Reference
*N* = 6,484, female = non specified	Olympic distance	Performance trends of age group athletes at the ITU World Championships over the Olympic distance triathlon from 2009 to 2014	• Women and men improved performance in most age groups across years.	(58)
*N* = 113, female = 58	Olympic distance	Changes in performance for both elite women and men in the ITU World Triathlon Series between 2009 and 2012	• Swimming and running split times remained unchanged whereas cycling split time and overall race times increased significantly for both women and men.	(228)
*N* = 7,939, female = 1,666	Olympic distance	Performance trends at the Olympic distance “Zürich Triathlon” from 2000 to 2010	• During this period, overall race performance time did not significantly change for both sexes except for the men in the age group 50–54 years and for the women in the age group 45–49 years who improved their overall race time.	(47)
*N* = 17,786, female = 1'847	IRONMAN® distance	Changes in performance in the IRONMAN® Switzerland from 1995 to 2010	• The cycling times and overall race times decreased whereas the swimming and running times showed no changes for both sexes. • The sex difference in performance in the three disciplines and for overall race time decreased significantly across years. • Women and men improved overall race times by approximately 1.2 and 4.2 min/year, respectively.	(226)
*N* = 410,881, female = 81,815	IRONMAN® distance	Performance of all pro and age group triathletes ranked in all IRONMAN® triathlons held worldwide between 2002 and 2015	• In pro athletes, performance improved in all disciplines. • Performance improved in younger age groups for running and older age groups for swimming and cycling.	(225)
*N* = 2,027, female 175	IRONMAN® distance	Performance trends in “Isklar Norseman Xtreme Triathlon” from 2003 to 2015	• Across years, women improved in swimming and both sexes improved in cycling and in overall race time. • In running, however, neither women nor men improved. • Overall race time decreased across years in both women and men.	(229)
*N* = 2,400, female = 1,200	IRONMAN® distance	Changes in performance of elite female and male triathletes in “IRONMAN® Hawaii” between 1983 and 2012	• Both female and male elite triathletes improved their performance in cycling, running and overall race time during the studied period.	(24)
Top 10 women and men between 1981 and 2007	IRONMAN® distance	Changes in performance in “IRONMAN® Hawaii” between 1981 and 2007	• Overall performance time of elite female and male triathletes decreased rapidly between 1981 and the late 1980s and then plateaued thereafter for both women and men.	(13)
*N* = 35,293, female = 7,825	IRONMAN® distance	Changes in performance of masters triathletes at the IRONMAN® triathlon World Championship from 1986 to 2010	• Female triathletes older than 40 years and male triathletes older than 44 years significantly improved their swimming, cycling, running and overall race time performances in the most recent years.	(37)
*N* = 904, female = 80	Triple Iron ultra-triathlon distance	Changes in performance in Triple Iron ultra-triathlon across years from 1988 to 2011	• Overall race time decreased for men, while it increased for women across years.	(11)
*N* = 42,961, female = 9,502	IRONMAN® to Double Deca Iron ultra-triathlon	Changes in performance in top performers for ultra-triathlon races held between 1978 and 2013	• Split and overall race times decreased nonlinearly across years in IRONMAN® triathletes. • In Triple Iron ultra-triathlon, overall race times increased nonlinearly in women. • For the other races, overall race times remained unchanged for women across years.	(230)
*N* = 5,049, female = 552	Double, Triple, Quintuple and Deca Iron ultra-triathlons	Trends in performance in ultra-triathlons from 1985 to 2018	• Performance analysis showed a negative trend over time for women and men since 1985. • Performance in ultra-triathlons has been decreasing in women and men over the years, but sex difference in performance remained.	(38)
*N* = 668, female = 98	“Ultraman® Hawaii” (day 1 with 10-km swimming and 145-km cycling, day 2 with 276-km cycling, and day 3 with 84-km running)	Changes in performance in “Ultraman® Hawaii” from 1983 to 2012	• Overall race times of the annual winners and the annual three fastest finishers decreased over time for both women and men.	(9)

ITU, International Triathlon Union.

#### 3.2.2 Sex difference in overall performance

Sex differences in performance vary across the different disciplines and distances in triathlon. (7) Table 4 shows the key findings of the studies investigating the sex difference in overall performance in different distances from Olympic distance to Double Deca ultra-triathlon. With the increase in female participation in recent years, the gap between sexes in performance seems to be generally narrowing (7, 10). Although there has been an improvement in female performance (73), there still exists a gap between the sexes (74).

**Table 4:** Overview of key findings from studies investigating sex differences in overall performance in different triathlon distances.

Subjects	Distance	Observation	Outcome	Reference
*N* = 6,484, female = non specified	Olympic distance	Changes in terms of sex difference of age group athletes at the ITU World Championships over the Olympic distance triathlon from 2009 to 2014	• The sex difference in performance between women and men remained constant.	(58)
*N* = 113, female = 58	Olympic distance	Investigation of the sex difference in performance in the ITU World Triathlon Series between 2009 and 2012	• The sex difference in performance decreased for overall race time from 11.9 ± 1.2% to 11.4 ± 1.4%. • The world's best female short-distance triathletes reduced the gap with male athletes in total performance at short distance triathlon with drafting during the 2009–2012 period.	(228)
*N* = 57, female = 18	Olympic distance	Sex difference in overall performance at a national Olympic triathlon	• The sex difference in performance was 8.0% for overall race time.	(71)
*N* = 7,939, female = 1,666	Olympic distance	Sex difference in performance at the Olympic distance “Zürich Triathlon” from 2000 to 2010	• For elite triathletes, the sex differences did not significantly change for the three disciplines and for overall race time. • The mean sex difference in overall race time was 14.8 ± 1.8% for the elite top 5 overall triathletes. • The sex difference in overall performance increased after the age of 35 years.	(47)
*N* = 540, female = 270	IRONMAN® distance	Sex difference in performance in IRONMAN® triathlon between 2006 and 2008	• The mean sex difference in overall race time was 15.8%.	(25)
*N* = 410,881, female = 81,815	IRONMAN® distance	Performance of all pro and age group triathletes ranked in all IRONMAN® triathlons held worldwide between 2002 and 2015	• The sex difference remained stable across years with minor exceptions, where women were able to reduce the sex difference.	(225)
*N* = 2,027, female = 175	IRONMAN® distance	Sex differences in performance in “Isklar Norseman Xtreme Triathlon” from 2003 to 2015	• For overall race time, no differences between women and men were found.	(229)
*N* = 687,696, female = 134,088	IRONMAN® distance	Sex difference in performance in IRONMAN® races between 2002 and 2022	• Men were faster than women in all groups for all split disciplines and overall race time.	(60)
*N* = 220, female = 110	IRONMAN® distance	Sex differences in performance time at the IRONMAN® triathlon World Championship 2018	• Independent of age, the averaged sex difference in overall performance time was 15.1 ± 3.6%.	(23)
*N* = 35,293, female = 7,825	IRONMAN® distance	Sex differences in performance as a function of age at the IRONMAN® triathlon World between 1986 and 2010	• Sex differences in IRONMAN® overall race times decreased between 1986 and 2010 for masters age groups while it remained stable for the young age group, 18–39 years.	(37)
*N* = 1,620, female = 810	Sprint, Olympic and IRONMAN® 70.3 distance	Sex differences in performance across age groups among varying triathlon distances in amateur triathletes from the 2009–2011 Triathlon World Championship	• Sex differences for total performance time were greatest in the youngest age groups and older age groups for Sprint, Olympic, and IRONMAN® 70.3 distances.	(75)
*N* = 20,638, female = 5,163	IRONMAN® and Double Iron ultra-triathlon distance	Sex difference in overall race times and split times between elite female and male IRONMAN® triathletes competing in “IRONMAN® Hawaii” and Double Iron ultra-triathletes between 1999 and 2011	• In “IRONMAN® Hawaii” and Double Iron ultra-triathlon, the sex difference in performance of the top three athletes remained unchanged for overall race time. • Sex differences increased as endurance race distances increased and showed no changes over time.	(231)
*N* = 3,057, female = 257	Double—Deca Iron ultra-triathlon distance	Sex differences in performance in ultra-triathlon between 1985 and 2005	• Concerning World best performances, the men were ∼19% faster than the women in both the Double and Triple Iron, and ∼30% faster in a Deca Iron. • With increasing length of ultra-triathlons, the best women became relatively slower compared with the best men.	(3)
*N* = 1,746, female = 155	Double Iron ultra-triathlon distance	Changes in sex difference in Double Iron ultra-triathlon race times between 1985 and 2012	• Men were faster than women for overall race times. • The sex difference remained unchanged over time for overall race time. • The sex differences for overall race times were higher than reported for IRONMAN® triathlon.	(56)
*N* = 904, female = 80	Triple Iron ultra-triathlon distance	Changes in sex difference in performance in Triple Iron ultra-triathlon across years from 1988 to 2011	• The gender difference in overall race time for winners increased from 10% in 1992 to 42% in 2011.	(11)
*N* = 46,123, female = 9,802	IRONMAN® to Double Deca Iron ultra-triathlon distance	Sex difference in ultra-triathlon performance in different triathlon distances between 1978 and 2013	• The fastest men were faster than the fastest women regarding overall race times. • Women reduced the sex difference in the IRONMAN® distances but extended the sex difference in longer distances (i.e., Double and Triple Iron ultra-triathlon).	(64)
*N* = 42,961, female = 9,502	IRONMAN® to Double Deca Iron ultra-triathlon distance	Changes in sex difference in top performers for ultra-triathlon races held between 1978 and 2013	• The sex difference in performance decreased in IRONMAN® and increased in Triple Iron ultra-triathlon but showed no changes across years for the other race distances.	(230)
*N* = 5,049, female = 552	Double Iron, Triple Iron, Quintuple Iron, and Deca Iron ultra-triathlons	Sex differences in different ultra-triathlon distances from 1985 to 2018	• Performance in ultra-triathlons has been decreasing in women and men over the years, but sex difference in performance remained.	(38)
*N* = 668, female = 98	“Ultraman® Hawaii” (day 1 with 10-km swimming and 145-km cycling, day 2 with 276-km cycling, and day 3 with 84-km running)	Changes in sex differences in overall performances in “Ultraman® Hawaii”	• The sex-related difference in performance decreased over time.	(9)

ITU, International triathlon union.

The sex gap in overall triathlon performance at the IRONMAN® distance in elite triathletes has been reduced to less than 10% thanks to significant improvements in marathon performance among women (63). Compared to the IRONMAN® distance, a recent study demonstrated that in the Olympic distance, an 8% sex difference in overall performance was observed among amateur athletes (71). Higher differences were evident when examining the top 10 amateur athletes at the World Championship from 2009 to 2011 (12% sex difference in overall race time) (75) and in an analysis of the “Zurich Triathlon” between 2000 and 2010 for the top 5 overall elite triathletes (14.8% in overall race time) (47). It should be noted that the comparison of sex-specific differences is complicated by the varying competition conditions at different events (63) and the diverse levels of performance (71).

When comparing the sex differences in the IRONMAN® performance with those in the Double or Triple Iron ultra-triathlon distances, discrepancies amplify with the length of the ultra-triathlon (3). While the sex difference remained consistent on the Double Iron ultra-triathlon distance over the years (56), there was an observable augmentation in the performance gap between sexes on the Triple Iron ultra-triathlon distance (11). Women became slower in the Triple Iron ultra-triathlon between 1988 and 2011, increasing in the sex difference from 10% in 1992 to 42% in 2011 (11).

Despite some authors questioning whether the gap between women and men can be closed (50–52), newer studies have not confirmed this and have shown that the sex difference in both endurance performances (53, 54) and anaerobic sprints (76) is no longer decreasing. It appears that the sex difference in performance has reached a plateau (53). This is also evidenced by an analysis of the “IRONMAN® Hawaii” between 1981 and 2007 (13).

#### 3.2.3 Sex differences in performance in the different split disciplines

Performance disparities between sexes are evident in the subdisciplines of triathlon (swimming, cycling, running) (63). For instance, at the IRONMAN® 70.3 and IRONMAN® distance, a smaller sex difference was observed in swimming compared to cycling and running across in age group athletes (6, 25, 63), while at the Olympic, the sex difference in cycling was smaller compared to swimming and running for both elite and age group athletes (47). Table 5 summarizes the key findings of studies investigating the sex differences in performance in the split disciplines.

**Table 5:** Overview of key findings from studies investigating sex differences in the split disciplines’ performance in different triathlon distances.

Subjects	Distance	Observation	Outcome	Reference
*N* = 113, female = 58	Olympic distance	Changes in sex difference in performance for the split discipline in the ITU World Triathlon Series between 2009 and 2012	• The sex difference in performance remained unchanged for swimming and cycling but decreased for running from 14.9 ± 2.7% to 13.2 ± 2.6%. • The sex difference in running was greater compared to swimming and cycling.	(228)
*N* = 57, female = 18	Olympic distance	Sex difference in performance in the split disciplines at a national Olympic triathlon	• The sex differences in performance were 11% for swimming, 7.5% for cycling, and 7% for running.	(71)
*N* = 7,939, female = 1,666	Olympic distance	Sex differences in performance in the split disciplines at the Olympic distance “Zürich Triathlon” from 2000 to 2010	• The mean sex differences in swimming, cycling and running time were 15.2 ± 4.6%, 13.4 ± 2.3% and 17.1 ± 2.5%, respectively. • For both elite and age group athletes, the sex difference in cycling time was significantly lower than for swimming and running.	(47)
*N* = 823,459, female = 198'066	IRONMAN® 70.3 distance	The sex differences in the split disciplines and overall race times by age group between 2004 and 2020	• Men were faster than women in all split disciplines and all age groups. • The sex difference was lower in swimming than in cycling and running and less pronounced for triathletes between the age 20 and 50 years. • The sex difference in swimming and running was U-shaped throughout age groups, with an increase after 18–24 years in swimming and after 40–44 years in running. In contrast, the sex difference in cycling decreased continuously with increasing age.	(6)
ND.	Olympic and IRONMAN® distance	Review of sex difference in triathlon performance	• For non-elite IRONMAN® triathletes, sex difference in swimming time (∼12%) is lower than for cycling (∼15%) and running (∼18%). • For elite triathletes, sex difference in running performance is greater for Olympic distance (∼14%) than for IRONMAN® distance (∼7%). • Elite female IRONMAN® triathletes reduced the gap to their male counterparts to less than 10% for the marathon.	(63)
Top 10 female and male between 1981 and 2007	IRONMAN® distance	Sex differences in the split disciplines, and overall performance in “IRONMAN® Hawaii” for the top 10 female and male finishers from each year between 1981 and 2007	• Female triathletes improved their running time by 7.9 min per decade since 1988. • The mean sex difference in time for swimming, cycling, running, and total event were 9.8%, 12.7%, 13.3%, and 12.6%, respectively.	(13)
*N* = 540, female = 270	IRONMAN® distance	Sex difference in swimming, cycling, and running performances in “IRONMAN® Hawaii” triathlon between 2006 and 2008	• Mean sex difference in performance time was significantly smaller for swimming (12.1 ± 1.9%) compared to cycling (15.4 ± 0.7%) and running (18.2 ± 1.3%).	(25)
*N* = 60, female = 30	IRONMAN® distance	Differences in split times in professional female and male IRONMAN® World Championship participants in the fastest IRONMAN® World Championship ever in 2022	• The top 10 women and men were faster in cycling and running than lower-placed athletes. • In contrast, the top 10 male athletes were slower in swimming than other groups.	(203)
*N* = 17,786, female = 1’847	IRONMAN® distance	Changes in sex differences in IRONMAN® Switzerland from 1995 to 2010	• The sex difference in running (18.2 ± 4.1%) performance was significantly greater compared with swimming (14.0 ± 5.4%) and cycling (13.2 ± 2.4%). • The sex difference in the split disciplines decreased significantly across the years.	(226)
*N* = 41,367, female = 7,983	IRONMAN® distance	Top ten performance of athletes in various age groups in “IRONMAN® Hawaii” and its qualifier races in 2010	• Swim split times were slower in “IRONMAN® Hawaii” than in the qualifier races for women and men in all age groups. • Cycling split times were slower in “IRONMAN® Hawaii” than in the qualifier races for women aged 18–24, 25–29, 40–44, 50–54 and 60–64 years. • Run split times were slower for women aged 18–24 years in “IRONMAN® Hawaii” than in the qualifier races.	(10)
*N* = 687,696, female = 134,088	IRONMAN® distance	Sex difference in the split disciplines	• The sex difference was more pronounced in cycling compared to swimming and running. • Between age group 35–39 years and 60–64 years, the sex difference was nearly identical in swimming and running. • For age groups 75 + years, the sex difference decreased in swimming and cycling but increased in running.	(60)
*N* = 2,027, female 175	IRONMAN® distance	Sex differences in performance in “Isklar Norseman Xtreme Triathlon” from 2003 to 2015	• Men were faster than women in cycling, but not in swimming, running and overall race time.	(229)
*N* = 220, female = 110	IRONMAN® distance	Sex differences in performance time at the 2018 IRONMAN® triathlon World Championship	• Independent of age, the averaged sex difference in swimming, cycling, running, and overall performance time was 14.0 ± 3.3%, 15.6 ± 3.1%, 15.3 ± 6.8% and 15.1 ± 3.6%, respectively.	(23)
*N* = 1,620, female = 810	Sprint, Olympic and IRONMAN® 70.3 distance	Sex differences across age groups for the different modes of locomotion among varying triathlon distances in amateur triathletes from the 2009–2011 triathlon World Championship	• Sex differences varied among the modes of locomotion for the three distances of triathlons. • However, for short- to mid-distance triathlons, performance time seem to indicate that the largest sex differences exist for swimming.	(75)
*N* = 1,746, female = 155	Double Iron ultra-triathlon	Changes in sex difference for the split disciplines in Double Iron ultra-triathlon between 1985 and 2012	• Men were faster than women for split times. • The sex difference remained unchanged over time for split times. • The sex differences for split times were higher than reported for IRONMAN® triathlon.	(56)
*N* = 46,123, female = 9,802	IRONMAN® to Double Deca Iron ultra-triathlon distance	Sex difference in ultra-triathlon performance in different triathlon distances between 1978 and 2013	• The sex difference in the swimming split increased with increasing race distance.	(64)
ND.	Different swimming distances (sprint to ultra-distances)	Sex differences for all swimming strokes, different distances, extreme conditions, different ages and swimming integrated in multi-sport disciplines	• In triathlon swimming performance, the sex gap remained stable during shorter and longer swim distances. In contrast, women can outperform men in events with extreme water temperatures. • Regarding open-water swimming, women seemed to continuously narrow the gap to men, especially in specific long-distances.	(84)
ND.	Different triathlon distances	Review of sex differences in triathlon performance	• Sex differences in triathlon performance differ generally between the locomotion modes, with lower differences seen in swimming than in cycling and running for both elite and non-elite triathletes.	(7)

ITU, International triathlon union.

The differences across triathlon distances can be attributed to factor such as pacing strategies (34), competition density (70), and variations in training and experience levels (22, 77). In contrast, a more significant sex difference in cycling compared to running and swimming was found in an investigation of the IRONMAN® races between 2002 and 2022 (60). The contrasting findings on sex differences in triathlon disciplines may be explained by methodological differences, course and environmental factors (78, 79), participation rates and competitive depth (70).

The following studies have separately focused on performance in ultra-distance swimming, ultra-cycling, and ultra-running separately. In terms of swimming performance, Knechtle et al. demonstrated that women can outperform men in ultra-swimming events such as the “Manhattan Island Marathon Swim” between 1983 and 2013. The best women were −12%–14% faster than the men in a 46-km open water ultradistance race with temperatures under 20 degrees (80). Other studies have similarly suggested that women can achieve comparable or even superior performance to men in ultra-swimming (81–83). A review by Knechtle et al. showed that women were ∼0.06 km/h faster than men in open-water long-distance swimming events of the “Triple Crown of Open Water Swimming” (“Catalina Channel Swim”, “English Channel Swim” and “Manhattan Island Marathon Swim”) (84). An explanation for this could be women's greater ability to metabolize fat, the better hydrodynamics, and the more even pacing strategy compared to men, which may be advantageous, especially during prolonged swimming competitions (85).

Several studies have also shown a significant decrease in the sex difference in open-water ultra-distance swimming performance over the last years (81, 83, 86, 87). Additionally, a decrease in sex differences with increasing distance has been noted in shorter events. Tanaka et al. demonstrated a gradual narrowing of the sex gap in swimming with extended distances. While the difference was 19% at 50 m, it decreased to 11% at 1,500 m (88). In contrast, other studies showed no changes in sex difference in short distance (89) and ultra-distance swimming (90) over the years.

In ultra-cycling, Baumgartner et al. showed that men were faster than women for race distances between 100 and 500 miles (169.9 km and 804.7 km) in most of the years from 1996 to 2018. The sex difference was greatest in 100-mile races, while it was able to be reduced in the longer distances (200-, 400-, and 500-mile races) (91).

Similar results have also been observed in ultra-running. According to a recent analysis of ultra-running, sex differences decreased with older age and longer distance, while in 100-mile races the difference was 4.41% and in 50-mile races 9.13% (92).

Between 1988 and 2007, women improved their Hawaii IRONMAN® marathon time by 0.8 min per year, while men's times remained constant (13). It appears that women have narrowed the gap to men the most in the marathon running during the IRONMAN® (7). From 1985 to 2004, women's marathon performance improved nearly threefold greater than the rate observed in men (53). Notably, the sex difference in the marathon run of “IRONMAN® Hawaii” aligns that of the “New York Marathon”, suggesting that swimming and cycling factors do not exacerbate the sex gap in running (13).

Nevertheless, women are unable to match men in ultra-running (93, 94) and ultra-cycling (95, 96). A study investigating performance and sex differences in ultra-triathlons between 1978 and 2013 for the three fastest finishers ever revealed that as the distance increases, sex differences in performance remained stable with increasing distance, except in the swimming split where the sex difference increased (64). In contrast to the studies mentioned earlier, this research demonstrated that the sex difference in swimming amplified with distance (64). One plausible explanation for this phenomenon is the athlete's background. Triathletes must compete in three different sports, and each athlete may have a unique background. Many triathletes might have more experience in running or cycling than in swimming (64). Considering the established correlation between performance in cycling and running with success in ultra-triathlons (97, 98) as well as in other distances (14, 99–101), athletes benefit from their experience in these areas in particular.

#### 3.2.4 Predictive variables for race performance

Research studies vary on which discipline is the optimal predictor of overall triathlon performance. There is disagreement as to whether cycling or running is the superior predictor of performance (2, 102, 103). Notably, in the Olympic distance, it has been determined that the running split emerges as the crucial determinant (14, 99). Conversely, Weiss et al. have recently illustrated that cycling demonstrated the strongest correlation with overall race time on the IRONMAN® 70.3 distance, followed by running (100). In a correlation analysis to verify the association level between the overall race time and the split time in IRONMAN® 70.3 age group triathletes have been showed that there were stronger associations of cycling and running with overall race time than swimming and a more negligible difference in swimming performance between women and men (104). A study examining 43 IRONMAN® triathletes (27 men, 16 women) revealed that among men, cycling exhibited the highest correlation with overall race time, whereas among women, running and cycling displayed approximately equivalent associations (101). However, no study has established swimming as a predictor of performance. The contribution of swimming split times to the overall race time is notably lower compared to cycling and running in ultra-triathlon (77).

Potential predictive variables for a fast race time for the IRONMAN® distance and ultra-triathlon have been analyzed. The most important predictive variables for a fast IRONMAN® race time were age of 30–35 years (women and men), a fast personal best time in the Olympic distance triathlon (women and men), a fast personal best time in marathon running (women and men), both a high volume and a high speed in training where high volume was more important than high speed (women and men), low body fat, low skin-fold thicknesses and low circumference of upper arm (only men), and origin from the United States of America (women and men) (15). Knechtle et al. also found that the origin of the athlete and the age group were the most important predictors in IRONMAN® races (105). For ultra-triathlon, the most important predictive variables were male sex, low body fat, age of 35–40 years, extensive previous experience, a fast time in cycling and running but not swimming, and origins in Central Europe (77).

To summarize this section, future studies need to consider the impact of the male-to-female ratio on participation, as this can influence competitive dynamics and the observed performance gap. Recent studies have shown that women are still underrepresented in research (106, 107). Whereas 10 years ago the proportion of women was 39%, today it has risen to 43.95% (48, 106). Additionally, in sports science, there are more male-only (31%) studies than female-only (6%) studies, leading to a knowledge gap in women-specific physiology and performance (107). This indicates that, despite progress, there remains a substantial need for further effort in research to achieve equitable sex representation. Future research should also account for advancements in technology over recent decades, such as improvements in equipment, racewear, and recovery tools, which may have affected the performance of female and male athletes differently. Furthermore, there is limited information on how environmental factors, such as the surrounding temperature, affect the performance in female and male triathletes. Therefore, future studies need to investigate the influence of the environmental conditions on triathlon performance.

### 3.3 Physiology, morphology, and anthropometry

There is consensus in the literature that maximal oxygen uptake (VO_2_max) and body composition are key variables associated with performance in longer triathlon distance events (108). It is also well-established that male athletes generally demonstrate superior aerobic performance in various endurance sports compared to female athletes (63, 71).

In Table 6, the key findings from studies examining physiological characteristics in female and male triathletes are summarized.

**Table 6:** Overview of key findings from studies investigating aspects of physiology in female and male triathletes.

Subjects	Distance	Observation	Outcome	Reference
*N* = 41, female = 19	Olympic distance	VT, RCP, and the percentage of MAS that can be maintained in a triathlon race	• VO_2_max and MAS were significantly higher in male athletes than in female athletes. • Female and male amateur athletes presented similar VT and RCP related to VO_2_max. • The speeds at VT and RCP were higher in male athletes. • Female athletes maintained the running split at a higher relative intensity considering the percentage of RCP than male athletes.	(27)
*N* = 57, female = 18	Olympic distance	VO_2_max, VT, and RCP of amateur triathletes	• Female athletes presented a lower VO_2_max and a higher %VO_2_max at VT and RCP than male athletes.	(71)
*N* = 57, female = 23	Olympic distance	Aerobic muscle quality, accessed by VO_2_ max and adjusted by lower limb lean mass, between women and men; and sex differences according to VO_2_ submaximal values assessed at VT	• Absolute and body mass-adjusted VO_2_max was higher in male athletes than in female athletes. • Absolute and body mass-adjusted VO_2_ at VT and RCP were higher in male athletes than in female athletes. • No sex difference in lean mass-adjusted VO_2_ max. • MAS and speeds at VT and RCP were higher in male athletes than in female athletes.	(120)
*N* = 45, female = 6	Olympic distance	Predictors of overall race time and disciplines in the Olympic distance triathlon	• MAV is part of all the prediction equations for performance in each discipline and in total race time. • The faster amateur triathletes were younger with superior body composition and aerobic capacity than their slower counterparts.	(17)
*N* = 40, female = 20	Olympic distance	Physiological and body composition variables between sexes	• Men had significantly higher values for VO_2_max, MAS, VT speed and RCP speed. • No significant difference in the percentage of VO_2_max at VT or RCP. • The overall race time could have been predicted by VO_2_max and lean mass in females.	(18)
*N* = 12, female = 12	No distance specified	Peak aerobic capacity in female triathletes in 3 modes of exercise: treadmill, cycle, and arm ergometer.	• Results indicated VO_2_ peak (ml/kg/min) is highest on a treadmill (46.8 ± 2.1), intermediate in cycling (40.7 ± 5.0), and lowest in arm ergometry (28.2 ± 3.3) with mean differences being significant (*p* ≤ 0.05).	(232)

VT, ventilatory threshold; RCP, respiratory compensation point; MAS, maximal aerobic speed; VO_2_max, maximal oxygen uptake; MAV, maximum aerobic velocity.

#### 3.3.1 Maximum oxygen uptake (Vo_2_max)

In literature, there is consensus that female athletes tend to exhibit relatively lower VO_2_max values (ml/min/kg) during cycling or running compared to their male counterparts (63, 71). The Table 7 summarizes the values of VO_2_max from studies that investigated physiological characteristics in triathletes. The mean values in these studies for VO_2_max were for women in Sprint distance 45.9 ± 2.5 ml/kg/min and in Olympic distance 50 ± 0.6 ml/kg/min, and for men 51.4 ± 1.0 ml/kg/min and 57.6 ± 3 ml/kg/min, respectively. Compared to a study, where 33 female IRONMAN® triathletes were measured, the values for VO_2_max in Sprint and Olympic distances were smaller (109). It should be noted that the sample sizes of these studies were small and the level of the participants and the used measurements were different.

**Table 7:** Values for VO_2_max and anaerobic threshold from studies investigating physiological variables.

Distance	Subjects	VO_2_ max (mL/kg/min)	Anaerobic threshold (%VO_2_ max)	Exercise modality	Measured threshold	Reference
Sprint distance	M = 21	52.1 ± 8.2	68.8 ± 6.2	Cycling ergometer	VT	(233)
	F = 17	44.1 ± 4.9	69.9 ± 6.3			
Sprint distance	M = 16	50.7 ± 8.1		Cycling ergometer		(26)
	F = 16	47.7 ± 7.0				
Olympic distance	M = 39	59.9 ± 6.3	74.4 ± 5.6	Treadmill	VT	(17)
	F = 6	50.3 ± 6.1	82.8 ± 5.0			
Olympic distance	M = 20	54.6 ± 5.0	73.8 ± 4.7	Treadmill	VT	(18)
	F = 20	49.7 ± 7.6	76.2 ± 5.3			
Olympic distance	M = 22	54.0 ± 5.1	74.4 ± 4.9	Treadmill	VT	(27)
	F = 19	49.8 ± 7.7	76.1 ± 5.4			
Olympic distance	M = 34	59.7 ± 5.8	74.7 (calculated)	Treadmill	VT	(120)
	F = 23	50.9 ± 6.9	77.5% (calculated)			
Olympic distance	M = 39	59.9 ± 6.3	74.4 ± 5.6	Treadmill	VT	(71)
	F = 18	49.5 ± 7.8	78.7 ± 6.1			
Different distances	*N* = 13, F = 6	56.3 ± 5.56	73.8 ± 5.18	Treadmill	VT	(234)
Different distances	M = 125	52 ± 6	∼74.6 (calculated)	Treadmill	VT	(235)
	F = 18	45.3 ± 4.9	∼79.5 (calculated)			
No distance specified	M = 11	56.0 ± 5.9		Cycling ergometer		(141)
	F = 11	47.7 ± 5.8				
No distance specified	F = 10	63.6 ± 1.2	74.0 ± 2.0	Treadmill	VT	(236)
No distance specified	M = 29	63.2 ± 4.5	85 ± 4.9	Treadmill	LT	(125)
	F = 15	55.4 ± 3.9	87.6 ± 6			

M, male participants; F, female participants; VT, ventilatory threshold; LT, lactate threshold.

VO_2_max is limited by the cardiovascular capacity to transport oxygen, in the vast majority of people (110), showing a strong relationship with total hemoglobin (111) and maximal stroke volume (112). The discrepancy of VO_2_max is frequently ascribed to central factors, such as women's smaller hearts and lower hemoglobin mass, which may limit their capacity to supply oxygen to skeletal muscles (113, 114). Further variables affecting VO_2_max include blood cell mass, and hematocrit (110). Typically, women present lower values for these parameters (115–117). When comparing women's hemoglobin levels with those of “age and race-matched” men, women consistently demonstrated 12% lower mean hemoglobin levels irrespective of iron status (111, 118). Women's VO_2_max values adjusted to body mass are typically ∼20%-25% lower than men's (119, 120). A recent study by Martins et al. found no significant difference between women and men in VO_2_max values when adjusted for lean mass, even at submaximal training intensities (120). This parameter reflects muscular aerobic capacity and is limited by peripheral conditions such as capillary density, mitochondrial content in muscles, and enzyme levels in mitochondria (121). Given the absence of disparities in VO_2_max values adjusted for lean mass between the sexes, it can be inferred that skeletal muscles in female and male athletes possess equivalent oxygen extraction capabilities (120). However, some studies have reported differences in VO_2_max values adjusted for lean mass (122, 123). The discrepancies in findings from previous studies may be attributed to variations in the level of physical conditioning among female and male participants, small sample sizes, the methodologies used to assess lean mass, and the specialization of event distance (e.g., focusing on training for Olympic or IRONMAN® distances) (19, 120).Given that the performance gaps between the sexes are smaller than the discrepancy in VO_2_max (124), other physiological factors significantly influence performance (120).

#### 3.3.2 Anaerobic threshold

In addition to VO_2_max as an important parameter for performance, the lactate threshold also correlates with endurance performance (124). In triathletes, female athletes showed higher anaerobic thresholds than male athletes (17, 71). Similar results were found for athletes in the Olympic distance triathlon race, where Fernandes et al. (2023) studied 41 triathletes (22 men and 19 women) showing that the female athletes run at a higher percentage of the ventilatory threshold than the male athletes (27). Values for the anaerobic threshold listed in Table 7 are based on studies examining physiological factors in triathletes. In the different studies, a cardiorespiratory maximal test on a treadmill or a cycling ergometer were performed to identify the ventilatory threshold. The ventilatory threshold was identified by an increase in the ventilatory equivalent for oxygen without a corresponding rise in the ventilatory equivalent for carbon dioxide along with an increase in the partial pressure of exhale oxygen. The mean values in these studies for ventilatory threshold were for women in Sprint distance 69.9 ± 6.3% and at the Olympic distance 78.3 ± 2.8%, and for men 68.8 ± 6.2% and 74.3 ± 0.3% of VO_2_max, respectively.

In most of the studies investigating anaerobic threshold values for ventilatory threshold were available. Quittmann et al. (2022) measured the blood lactate in 24 runners and 20 triathletes with a total of 15 female and 29 male participants. They found a higher fractional utilization of VO_2_max at lactate threshold according to a fixed lactate concentration of 4 mmol/L (onset of blood lactate accumulation) in women (125).

A key factor in achieving a high lactate threshold is the capacity of mitochondria in muscles to increase their volume in response to training (126). Endurance training has been demonstrated to enhance lactate threshold (127, 128). Importantly, lactate threshold has been shown to correlate significantly with performance in prolonged running events like the marathon (124). Elite athletes can sustain 80%–90% of their VO_2_max for prolonged periods with a minor rise in blood lactate (124). Similar findings to triathletes were found in runners. In a study involving 75 long-distance runners (37 men and 38 women), slightly higher lactate thresholds (2.5%) were found in women than in men (129).

#### 3.3.3 Body composition

Moreover, body composition significantly impacts endurance performance. Body fat percentage has emerged as a crucial predictive factor in events such as the IRONMAN® 70.3 (16), IRONMAN® (21, 77), and ultra-triathlon distances (77). Table 8 shows the key findings of the studies investigating the aspects of body composition in female and male triathletes.

**Table 8:** Overview of key findings from studies investigating aspects of body composition in female and male triathletes.

Subjects	Distance	Observation	Outcome	Reference
*N* = 57, female = 18	Olympic distance	Fat mass and lean mass of amateur triathletes; measured with DEXA	• Female athletes presented lower lean mass than males. • Female athletes presented higher total fat mass and gynoid fat mass than men, but the same android and trunk fat masses.	(71)
*N* = 45, female = 6	Olympic distance	Predictors of overall race time and disciplines in the Olympic distance triathlon; measured with DEXA	• %LM is a predictor of cycle and total race time. • The faster amateur triathletes were younger with superior body composition and aerobic capacity than their slower counterparts.	(17)
*N* = 34, female = 4	IRONMAN® 70.3 distance	Physiological variables before and after an IRONMAN® 70.3 triathlon in the heat	• Post-race body mass change was positively correlated with race time while core temperature was negatively correlated with race time. • Post-race blood CK and myoglobin concentrations positively correlated with race time, suggesting that muscle breakdown is a possible cause for reduced performance in a triathlon.	(237)
*N* = 16, female = 16	IRONMAN® distance	Body mass, body fat, skeletal muscle mass, and hydration status; using the anthropometric method with a skin-fold callipser	• An IRONMAN® triathlon does not lead to a change in body mass in female triathletes, although the increase in urinary specific gravity might indicate dehydration.	(238)
*N* = 43, female = 16	IRONMAN® distance	Association between body fat, training volume, previous race experience and race performance in female and male triathletes; using the anthropometric method with a skin-fold callipser	• Percent body fat was associated only with men's total race time, whereas average weekly training time was related to women's performance. • For women and men, percent body fat and amount of training time were not related.	(22)
*N* = 43, female = 16	IRONMAN® distance	Association between skin-fold thickness and race performance in female and male IRONMAN® triathletes; using the anthropometric method with a skin-fold callipser	• In men, percent body fat, the sum of upper body skin-folds and the sum of all 8 skin-folds were related to total race time. • In women, none of the skin-fold thicknesses showed an association with total race time, average weekly training volume or speed in the sub disciplines in the race.	(101)
*N* = 43, female = 16	IRONMAN® distance	Association between anthropometric and training variables with race performance in an IRONMAN®;using the anthropometric method with a skin-fold callipser	• The anthropometry and training volume were differentially correlated to total race time in both female and male triathletes. • The percentage of body fat was not associated with training volume for both genders.	(21)
*N* = 93, female = 34	No distance specified	Comparison of several anthropometric parameters of professional elite triathletes with professional cyclists and sportive students	• The body weight and BMI are significantly lower in female and male triathletes than in the control groups.	(20)
*N* = 42, female = 42	No distance specified	Comparison of the absolute BMD and BMD adjusted to BSA between triathletes and healthy inactive women; measured with DEXA	• Triathletes had lower body mass and lower absolute BMD than nonactive women. • Triathletes had lower BSA than nonactive women. • Triathletes exhibited higher BMD adjusted to BSA than nonactive women.	(239)

%LM, percentage of lean mass; CK, creatine kinase; BMI, body mass index; BMD, bone mineral density; BSA, body surface area; DEXA, dual energy x-ray absorptiometry.

Regarding the association between body fat percentage and race performance, Knechtle et al. found a correlation between body fat percentage and overall race time in IRONMAN® distance events, but only in male participants (22). This observation was consistent with findings from another study (21). However, no significant correlation between anthropometric measures and performance was identified in Triple Iron (97, 98) and Deca Iron ultra-triathlon (130) events. It is worth noting, however, that these studies focused exclusively on male athletes.

Sex differences in body composition are notable among IRONMAN® triathletes, with men exhibiting an average body fat percentage of 14%, compared to 23% in female counterparts, measured with the anthropometric method using the skin-fold calliper (21). In addition to their lower body fat percentage, men typically exhibit greater muscle mass and muscle strength compared to women (21). In 27 male and 16 female IRONMAN® triathletes, it was shown that men had 41 kg of muscle mass, while women showed a muscle mass of 28 kg (21). Furthermore, women generally have lower body mass and height (71), although no discrepancies were observed in trunk fat percentage. However, women showed a higher proportion of gynoid fat mass (71). Table 9 summarizes the values of body mass in kilograms and body fat percentage from studies investigating anthropometric variables in triathletes. The values differ between the length of the race. The mean body weight (63.7 ± 0.2 kg in Sprint vs. 59.2 ± 0.9 kg in Olympic, 60.2 ± 0.8 kg in IRONMAN®) and the body fat percentage (28.1 ± 0.1% in Sprint vs. 22.8 ± 0.6% in Olympic, 23.1 ± 0.5% in the IRONMAN®) in women was greatest in Sprint distance. It must be noted that different methods were used to asses body composition, with DEXA (Dual Energy x-ray Absorptiometry) applied for sprint and Olympic distances, and anthropometric with a skin-fold calliper in the studies investigating the IRONMAN® distance. In each length, the mean values for body weight in women were smaller compared to men and the body fat percentage was higher. However, it is difficult to compare the studies as the level of the participants was different, the sample sizes were small, and varying methods of body composition measurement were used.

**Table 9:** Values for body mass, and body fat percentage from studies investigating anthropometric variables.

Distance	Subjects	Body mass (kg)	Body fat percentage (%)	Measuring method	Reference
Sprint distance	M = 21	77.1 ± 9.5	17.4 ± 6.0	DEXA	(233)
	F = 17	63.5 ± 6.6	28.2 ± 5.8		
Sprint distance	M = 16	76.1 ± 7.9	17.1 ± 6.2	DEXA	(26)
	F = 16	63.8 ± 6.6	28.0 ± 6.0		
Olympic distance	M = 39	74.3 ± 8.8	16.8 ± 5.6	DEXA	(17)
	F = 6	60.3 ± 5.1	22.7 ± 10.3		
Olympic distance	M = 20	74.8 ± 6.9	17.8 ± 6.3	DEXA	(18)
	F = 20	58.8 ± 6.7	23.3 ± 11.3		
Olympic distance	M = 39	74.3 ± 8.8	16.8 ± 5.6	DEXA	(71)
	F = 18	59.5 ± 5.6	23.2 ± 9.2		
Olympic distance	M = 34	74.9 ± 9.1	16.2 ± 5.3	DEXA	(120)
	F = 23	58.1 ± 6.6	21.9 ± 8.6		
IRONMAN® distance	M = 27	77.8	14.4	Anthropometric (skin-fold calliper)	(22)
	F = 16	59.8	22.8		
IRONMAN® distance	M = 27	75.8	13.7	Anthropometric (skin-fold calliper)	(21)
	F = 16	61.1	23.6		
IRONMAN® distance	F = 16	59.8	22.8	Anthropometric (skin-fold calliper)	(238)
Different distances	*N* = 13, F = 6	66.5 ± 12.6	17.7 ± 5.9	DEXA	(234)
Different distances	M = 125	79	15.4	Bioimpedance analyzer	(235)
	F = 18	61.9	23.3		
No distance specified	M = 11	75.2 ± 4.3	10.9 ± 6.6	Bioimpedance analyzer	(141)
	F = 11	57.2 ± 6.3	15.6 ± 6.8		
No distance specified	M = 27 F = 16	77.7 59.7	14.4 22.8	Anthropometric (skin-fold calliper)	(101)
No distance specified	M = 29	70.8 ± 7.2	11.9 ± 1.7	Anthropometric (skin-fold calliper)	(125)
	F = 15	58 ± 6.4	13.6 ± 4.9		
No distance specified	F = 23	57.6 ± 6.3	13.1 ± 7.0	DEXA	(239)

(mean value and standard deviation). M, male; F, female; N, numbers; DEXA, dual energy x-ray absorptiometry.

The higher body fat percentage in women can confer advantages in the swimming discipline, as it increases buoyancy in water (63), potentially contributing to the smaller sex difference observed in this discipline (131). Body fat might be related to greater substrate efficiency in ultra-endurance sports, where a prolonged exercise would induce the recruitment of fat stores (132). Moreover, body fat distribution is associated with performance (133), with higher android fat percentage reducing buoyancy and swimming speed (134). Additionally, increased body fat percentage provides better insolation in cold water (135), benefiting women's swimming performance, particularly in open-water long-distance swimming in cold temperatures (135, 136). However, during a 1.5 km swimming distance with a water temperature of around 20°C, women's higher body fat percentage does not confer advantages (47).

In addition to the lower muscle mass in female triathletes (21), studies indicated that women experience the loss of muscle mass and strength at an earlier stage than men (53, 137). This observation agreed with those in general population, where women had shorter limb levers, weaker bones and less muscle mass and muscle strength (138).

Research has also shown that women exhibit lower levels of muscular fatigue and faster recovery during endurance training (113). In controlled studies, it has been shown that women generally exhibit greater fatigue resistance than their male counterparts (139, 140). Even during high-intensity interval training (HIIT), Hottenrott et al. suggested that women may demonstrate greater fatigue resistance and enhanced metabolic recovery (141). This lower fatigue may be attributed to the higher proportion of type 1 muscle fibers in women (142).

To conclude this section, future studies investigating VO_2_max should adjust measurements to lean body mass, ensuring more accurate comparisons between female and male athletes. These studies would benefit from larger sample sizes and should include athletes of comparable skill levels, using standardized measurement protocols to ensure consistency. Additionally, future studies need to investigate running economy in female triathletes, as it was found to be a determinant of performance (143). Given known sex differences in fatigue resistance, biomechanics, and substrate utilization, research in this area could provide important insights for optimizing performance and training strategies for triathletes. Furthermore, there are no consistent findings on the performance during the menstrual cycle in other endurance sports (144–146). In triathlon-specific research, the effects of the menstrual cycle on triathlon performance are still scarce. Given the unique demands of the sport, further research is needed to understand how different menstrual cycle phases impact performance, recovery, and training in female triathletes. Understanding hormonal influences could help optimize training and race strategies for women in triathletes.

### 3.4 Aspect of age

The literature search identified a total of 20 studies that focused on age in triathletes. The Table 10 presents the key findings of studies investigating the effect of age on performance in triathlon and the age of peak performance in female and male triathletes. It is widely acknowledged that physiological, morphological, and functional capacities change with age (147). Furthermore, sex differences in endurance performance alter with increasing age (6, 7, 104). Multiple studies provide evidence of age-related effects on performance. Therefore, comprehending the age at which peak performance is achievable holds significant importance for athletes and their coaches regarding career strategizing (148).

**Table 10:** Overview of key findings from studies investigating the effect of age on performance in triathlon and the age of peak performance in female and male triathletes.

Subjects	Distance	Observation	Outcome	Reference
*N* = 7,939, female = 1,666	Olympic distance	Age and sex interaction at the Olympic distance “Zürich Triathlon” from 2000 to 2010	• For both sexes, overall race performance time did not significantly change except for the men in the age group 50–54 years and for the women in the age group 45–49 years that improved their total race time.	(47)
*N* = 6,303, female = 1,115	IRONMAN® 70.3 distance	Age-related differences in performance and sex differences at the IRONMAN® 70.3 Switzerland in Rapperswil, from 2007 to 2010	• The best performances across all split times and overall race time were achieved by triathletes falling between the ages of 25 and 39 years for women and between 18 and 39 years for men. • The sex difference in total time differed across age groups and between the locomotion mode.	(227)
*N* = 1,620, female = 810	Sprint, Olympic and IRONMAN® 70.3 distance	Best performance times across age groups for the different modes of locomotion among varying triathlon distances in amateur triathletes from the triathlon World Championship between 2009 and 2011	• In Sprint distance, the best performance times for women were seen in the 18–24 age group for swimming and the 30–34 age group in cycling, running, and total time. • In Olympic distance, the best performance time in women for swimming, running, and total time was in the 25–29 age group and for cycling in the 30–34 age group. • In IRONMAN® 70.3, the best performance time for women was in the 25–29 age group for all modes of locomotion, including total time.	(75)
*N* = 7,214, female = 2,942	Olympic, IRONMAN® 70.3 and IRONMAN® distance	Age of peak performance for world class athletes competing in Olympic, IRONMAN® 70.3 and IRONMAN® distance races, and potential change in the age of the annual fastest athletes between 2003 and 2013	• For the ten fastest women, the age of peak performance was significantly higher in athletes competing in IRONMAN® 70.3 and IRONMAN® distance compared to athletes competing in Olympic distance. • The age of the annual ten fastest women and men competing in IRONMAN® 70.3 and IRONMAN® distance remained unchanged. In Olympic distance, the age of the annual ten fastest women remained unchanged, while it decreased linearly in men.	(148)
*N* = 19,389, female = 2,051	IRONMAN® distance	Age of peak performance in swimming, cycling, and running, as well as overall race time for both elite female and male IRONMAN® triathletes from 1995 to 2011	• The mean age of the elite IRONMAN® triathletes was 33 ± 3 years for men and 34 ± 4 years for women. • For women, the age of peak performance was not significantly different between the three disciplines, while for men, the best swimmers (29 ± 3 years) were significantly younger than the best runners (35 ± 5 years). • The age of peak performance remained unchanged for men at 31 ± 3 years but increased for women from 30 ± 4 years in 1995 to 36 ± 5 years in 2011	(72)
*N* = 17,786, female = 1,847	IRONMAN® distance	Sex differences in age of peak performance in qualifier races for “IRONMAN® Hawaii” and “IRONMAN® Hawaii”; and changes in age of peak performance in IRONMAN® Switzerland between 1995 and 2010	• For all studied 20 races, the age of peak performance in IRONMAN® was 32.2 ± 1.5 years for men and 33.0 ± 1.6 years for women. • The age of peak performance in IRONMAN® showed no change across years although IRONMAN® race times became faster for both women and men across years.	(226)
*N* = 2,400, female = 1,200	IRONMAN® distance	Changes in age of elite female and male triathletes in “IRONMAN® Hawaii” between 1983 and 2012	• The age of the annual overall top ten triathletes increased for both women and men. • For men, the age increased from 27 ± 2 to 34 ± 3 years, and for women from 26 ± 5 to 35 ± 5 years.	(24)
*N* = 1,881, female = 879	IRONMAN® distance	Performance in different age groups in “IRONMAN® Hawaii” between 2003 and 2019	• The best overall race times were found in the 25–29, 30–34, 35–39, and 40–44 age groups for women and men. • No significant difference in mean race times were found between the 25–29, 30–34, 35–39, and 40–44 age groups of women and men.	(153)
*N* = 687,696, female = 134,088	IRONMAN® distance	Performance in different age groups in IRONMAN® races between 2002 and 2022	• The majority of the finishers were in the age group 40–44 years. • The age group 25–29 years had the fastest women, and the fastest men were in the age group 30–34 years.	(60)
ND.	IRONMAN® distance	Potential predictor variables for IRONMAN® race performance in female and male triathletes	• Among many other factors, an age of 30–35 years (women and men) is considered one of the most important predictive variables for a fast IRONMAN® race time.	(15)
*N* = 35,293, female = 7,825	IRONMAN® distance	Changes in performance of masters triathletes at the IRONMAN® triathlon World Championship from 1986 to 2010	• Female triathletes older than 40 years and male triathletes older than 44 years significantly improved their swimming, cycling, running and total time performances in the most recent years.	(37)
ND.	Ultra-triathlon distance	Potential predictive variables for a fast race time in an ultra-triathlon race from Double Iron ultra-triathlon and longer	• Among many other factors, an age of 35–40 years is one of the most important predictive variables for a fast race time in an ultra- triathlon from Double Iron and longer.	(77)

#### 3.4.1 Age of peak performance

The age of peak performance in IRONMAN® triathlons is between 25 and 39 years for both women and men (13, 25, 72). Knechtle et al. investigated the specific ages associated with peak performance across various distances (148). They determined that for women, the age of peak performance was 26.6 ± 4.4 years in the Olympic distance, 31.6 ± 3.4 years in the IRONMAN® 70.3 distance, and 34.4 ± 4.4 years in the IRONMAN® distance. The age of the annual top 10 women and men remained unchanged over the period from 2003 to 2013 in the IRONMAN® 70.3 and IRONMAN® distances (148). However, a study of the “IRONMAN® Hawaii” from 1983 to 2012 revealed a trend of increasing age among the annual top 10 performers alongside performance improvement. For men, the age of the annual top 10 increased from 27 ± 2 to 34 ± 3 years, and for women from 26 ± 5 to 35 ± 5 years (24). Overall, it seems that the age of peak triathlon performances increases with increasing race distance from the Olympic distance to IRONMAN® distance.

Regarding distances longer than the IRONMAN® distance, it was observed that the average age of finishers in the Deca Iron ultra-triathlon is notably higher than in the Triple Iron ultra-triathlon. Furthermore, the average age of finishers increased from 1992 to 2010 in both ultra-distances (149). Similarly, in ultra-marathons, it has been demonstrated that the age at which peak performance is attained is higher for longer distances (73, 74, 150, 151).

#### 3.4.2 Age-related performance decline

As illustrated in the preceding section, there has been a notable rise in the number of master athletes. Alongside this surge in participation among master athletes, remarkable improvements in performance have been observed, while the performance of athletes under the age of 40 years has remained relatively static (24, 37, 47, 152). It appears that athletes in younger age brackets have reached a plateau in performance across the IRONMAN® distance (24, 37). However, performance begins to decline with age (37, 137).

Table 11 summarizes the key findings of studies investigating the age-related performance decline in female and male triathletes.

**Table 11:** Overview of key findings from studies investigating the age-related performance decline in female and male triathletes.

Subjects	Distance	Observation	Outcome	**Reference**
*N* = 6,303, female = 1,115	IRONMAN® 70.3 distance	Age-related changes in performance at IRONMAN® 70.3 Switzerland from 2007 to 2010	• The age-related differences in IRONMAN® 70.3 performances differed between female and male athletes. • For both women and men, there was a reduced age-related decline in cycling performance when compared with running and swimming performance.	(227)
*N* = 343,345, female = 69,060	IRONMAN® distance	Effect of age on pacing of IRONMAN® triathletes	• The younger age groups were relatively faster in swimming, running and transition time, but relatively slower in cycling.	(36)
*N* = 410,881, female = 81,815	IRONMAN® distance	Age-related performance decline of all pro and age group triathletes ranked in all IRONMAN® triathlons held worldwide between 2002 and 2015	• Performance improved in younger age groups for running and older age groups for swimming and cycling. • The age-related decline in performance started at 25–29 years in swimming for women and men and in cycling, running and overall race time at 30–34 years and 35–39 years for women and men, respectively.	(225)
*N* = 1,881, female = 879	IRONMAN® distance	Age-related changes in performance in “IRONMAN® Hawaii” between 2003 and 2019	• After the age of 45–49 years, the overall race times increased with a significant difference in each age decade in both women and men.	(153)
ND.	Different triathlon distances	Review of age-related declines in triathlon performance	• Age-related declines in triathlon performance depend on the locomotion mode, the exercise duration, and the triathlon format (off-road vs. road based).	(7)

Puccinelli et al. have recently shown that female amateur triathletes in the “IRONMAN® Hawaii” present a similar pattern in age-related performance decline as their male counterparts. No differences in overall race time were found in the age groups from 25 to 29 years until 40–44 years. Female and male triathletes broke this plateau trend in the 45–49 age group, and the times for older age groups increased progressively (153).

In triathlon, the age-related performance decline appears to be contingent on the discipline involved (1, 149). Cycling showed a lesser decline compared to swimming and running (154). This discrepancy can be explained by the physiological and mechanical differences between cycling, running, and swimming, such as the transition from a weightless to a weight-bearing activity and the shift from a concentric muscle action in cycling to a stretching and shortening activity with eccentric contractions in running (155). Additionally, differences in training stimuli may explain the lower age-related performance decline in cycling compared to running and swimming (154, 155). Furthermore, it has been observed that the extent of the age-related decline varies depending on the race distance, with a more significant decline in cycling and running performance observed in the IRONMAN® distance compared to the Olympic distance (154).

Potential explanations for the performance improvements observed in master athletes may include increased participation rates, expanded training opportunities tailored to older individuals and a heightened competitive drive (37). Furthermore, analogous performance improvements have been observed among master athletes in other sporting domains, such as swimming (156) and ultra-marathon running (157).

The age-related decline in performance was also demonstrated in other endurance sport, for example in swimming (88, 158) and running (46). This decline typically became noticeable around the ages of 40–50 years, exhibiting a moderate progression until the age of 70, followed by an exponential decrease in endurance performance (46, 88, 158). Various factors contribute to the age-related decline in endurance performance. Age serves as a limiting factor for VO_2_max (159, 160). The progressive reduction in VO_2_max is the primary mechanism driving performance decline with age (161). Additionally, there is a concurrent loss of muscle mass with aging, which tends to occur faster in women than men (162, 163).

Further reasons for the more significant decline in performance with increasing age may encompass lifestyle adjustments, alterations in training regimens characterized by reduced volume and intensity, and dietary considerations among older athletes (147, 161, 164–167). The natural aging process can either be hastened or decelerated by lifestyle choices. The training status of master athletes emerges as a critical modulator of performance deterioration with advancing age. Changes in physiological functions and running performance with age are closely related to the extent of running training (167).

#### 3.4.3 Changes in sex differences in performance with age

An increase in sex differences with age at the IRONMAN® distance is evident. In Table 12, the key findings of studies investigating the changes in sex differences in performance with age in female and male triathletes are summarized. Lepers & Maffiuletti demonstrated that sex gaps in overall performance significantly widened with increasing age, particularly beyond 55 years of age, while remaining relatively stable before the age of 55 (25). Male athletes aged 60 exhibited a 27% slower pace than triathletes aged 30–40 years, whereas the difference for women was 38% (25). Additionally, Gries et al. showed an increase of the sex difference to 20% in the age group of 65-69 years (168).

**Table 12:** Overview of key findings from studies investigating the changes in sex differences in performance with age in female and male triathletes.

Subjects	Distance	Observation	Outcome	Reference
*N* = 1,620, female = 810	Sprint, Olympic and IRONMAN® 70.3 distance	Comparison of sex differences across age groups for the different modes of locomotion among varying triathlon distances in amateur triathletes from the 2009–2011 triathlon World Championship	• Sex differences in overall time were largest in 55–59-year age groups for Sprint and in 60–64-year age group for Olympic and IRONMAN® 70.3.	(75)
*N* = 823,459, female = 198,066	IRONMAN® 70.3 distance	Age-related performance decline in IRONMAN® 70.3 races between 2004 and 2020	• There was a progressive reduction of the sex difference in performance in age groups 50 years and older.	(104)
*N* = 823,459, female = 198,066	IRONMAN® 70.3 distance	Sex differences in the split disciplines and overall race times by age group in IRONMAN® 70.3 races between 2004 and 2020	• After the age of 60 years, women were able to reduce the sex difference to men in swimming and cycling, but not in running, where the reduction in the sex difference started after the age of 70 years. • The lowest sex difference was in the age group 75 + years for swimming and cycling and in the age group 30–34 years for running. • Across age groups, the sex difference was U-shaped in swimming and running, with an increase after 18–24 years in swimming and after 40–44 years in running. • The sex difference decreased continuously with increasing age for cycling.	(6)
*N* = 35,293, female = 7,825	IRONMAN® distance	Changes in sex differences in performance of master triathletes at the IRONMAN® triathlon World Championship from 1986 to 2010	• Sex differences in overall race time decreased for masters age groups while it remained stable for the young age group (18–39 years).	(37)
*N* = 687,696, female = 134,088	IRONMAN® distance	Changes in sex difference with age in IRONMAN® races between 2002 and 2022	• In the age group 18–24 years, the sex difference was lowest for all split disciplines and increased in a U-shaped manner until age group 70–74 years.	(60)
*N* = 540, female = 270	IRONMAN® distance	The effect of age on sex difference in performance in “IRONMAN® Hawaii” between 2006 and 2008	• The sex differences in overall race time were stable until 55 years of age after which they significantly increased.	(25)
*N* = 220, female = 110	IRONMAN® distance	Effect of age on the sex differences in performance time at the IRONMAN® triathlon World Championship 2018	• There was no significant change with age in the sex difference in performance for swimming. • For both cycling and running, the sex difference in performance of the age groups whose athletes were older than 60 years were significantly greater than those of younger age groups.	(23)
ND.	Different triathlon distances	Review of sex-related differences in triathlon performance with age	• Sex-related differences increase with advancing age most likely due to physiological, sociological, and psychological changes.	(7)

Explanations for this increase may encompass diverse physiological influencing factors (25) and the earlier onset of muscle mass loss in women (162, 163). Lepers et al. also noted the diminished participation of women in older age cohorts, which could contribute to a heightened sex difference (25). Moreover, hormonal differences pre- and post-menopause could further explain the more conspicuous age-related decline in performance among women (169). In addition to the pre- and post-menopause changes, female triathletes may also discover barriers that prevent them from returning to an exercise routine, including muscle weakness, fatigue, depression, and physical changes (170). The postpartum period is influenced by the number of births, the interval between births, the type and duration of labor and delivery as well as the length of time spent breastfeeding (170).

Future studies should explore the impact of pregnancy and childbirth on female triathletes, particularly how prolonged break from training affects recovery and fitness return. Additionally, research is needed on menopause-related hormonal changes and their effects on endurance, muscle function, and recovery, to develop training strategies tailored to aging female athletes.

### 3.5 Training, experience, pacing, nutrition and motivation

#### 3.5.1 Training and experience

Training (22, 171) and experience in triathlon (15, 77, 172, 173) are central factors influencing performance in triathlon competitions. The main conclusions of the studies examining the elements of experience and training in female and male triathletes are compiled in Table 13.

**Table 13:** Overview of the key findings from studies investigating the aspects of training and experience in female and male triathletes.

Subjects	Distance	Observation	Outcome	**Reference**
*N* = 9, female = 4	Olympic distance	Training characteristics of recreational-level triathletes within the competition period	• No changes in training loads, duration or training intensity distribution were seen in the weeks leading up to the competition. • Training duration was significantly reduced in week 6, while the number of sessions was reduced in week 6 and week 7.	(174)
*N* = 45, female = 6	Olympic distance	Predictors of overall race time and disciplines in Olympic distance triathlon	• Triathlon experience predicts swim, cycle, and overall race time.	(17)
*N* = 401, female = 194	Different distances (from Sprint to IRONMAN® distance)	Relationship between preparation strategies (training, mental preparation) and sexes	• Most participants engaged in strength training, consumed food, and/or fluids during and after training, set training and competition goals, and applied mental preparation strategies during training and the hour before racing. • Positive self-talk was the most used mental strategy. • Women were more likely than men to train with others, use mental preparation strategies, and report feeling anxious before competitions. • More men reported using nutritional supplements during training than their female counterparts.	(31)
*N* = 99, female = 19	IRONMAN® distance	Association between training volume and previous triathlon experience with overall and split race times	• Overall race time did not differ among those who trained up to 14 h per week, between 15 and 20 h per week or more than 20 h per week. • Triathletes who had a previous experience in IRONMAN® races achieved a better performance than those without previous experience.	(173)
*N* = 43, female = 16	IRONMAN® distance	Association between body fat, training volume, and/or previous race experience with female and male triathletes’ performance	• Female and male athletes’ best time in a previous triathlon was related to current IRONMAN® race performance. • Average weekly training time was related to women's performance. • For women and men, percent body fat and amount of training time were not related.	(22)
*N* = 9, female = 3	IRONMAN® distance	Training loads during an IRONMAN® training program based on intensity zones and training-performance relationships	• While athletes perform with heart rate mainly in zone 2, better performances are associated with more training time spent in zone 1. • A high amount of cycling training in zone 2 may contribute to poorer overall performance.	(180)
*N* = 43, female = 16,	IRONMAN® distance	Association between anthropometric and training variables with race performance in recreational female and male IRONMAN® triathletes	• The anthropometry and training volume were differentially correlated to overall race time in both female and male triathletes. • The percentage of body fat was not associated with training volume for both sexes.	(21)
*N* = 390, female = 224	IRONMAN® distance	Strength training and endurance training in long-distance triathletes	• Mean training hours per week was 14.92 ± 5.25, with 54.6% reporting participation in strength training. • Heavy strength training was the most commonly reported (39.4%), with significantly more men completing this form of strength training.	(30)
*N* = 1,971 (double), 1,075 (triple), 54 (quintuple), 131 (deca)	Different ultra- triathlon distances	Influence of the number of previously completed races and the personal best times in shorter races	• The number of finished shorter races was not associated with the number of finished longer races. • Split and overall race times correlated to split and overall race times of longer races except for the swimming split times in Double Iron ultra-triathlon, which showed no correlation with swimming split times in both Quintuple and Deca Iron ultra-triathlon.	(172)
*N* = 34, female = 11	No specific distance	Medical history, training regimen, and injuries in recreational triathletes	• Injury rates were higher in athletes who had completed a longer race and those who reported higher training times per week. • Additionally, many individuals had medical problems, used a variety of supplements, and followed specific dietary restrictions.	(28)
*N* = 16, female = 8	No specific distance	Changes in physiological and performance variables in triathletes following a 4-week period of reduced training volume and increased training intensity	• VO_2_max increased significantly in the HIIT group but remained unchanged in the control group. • Cycling power at first and second ventilatory thresholds increased significantly in the HIIT subjects and was unchanged in the control group participants. • No significant interactive effects between groups were observed for running time.	(29)

HIIT, high-intensity interval training.

##### 3.5.1.1 Training

In a study with 27 male and 16 female nonprofessional IRONMAN® triathletes, the weekly average training volume was 14.8 ± 3.2 h for men and 13.9 ± 3.4 h for women (22). The most training was spent in cycling with 8.0 ± 2.0 h for men and 7.5 ± 2.4 h in women (22). Similar results were found in 166 male and 224 female long-distance triathletes with a weekly training volume of 16 ± 4.9 h in men and 13.6 ± 5.5 h in women (30). In contrast, for Olympic distance triathlon the training volume per week was 8 h and 48 min (mean for 6 male and 5 female recreational-level triathletes) (174).

Weekly training duration impacts IRONMAN® performance differently across sexes. Research indicates that while weekly training volume significantly correlates with the total race time in women, this association is not observed in men (22, 101). Interestingly, neither sex showed a correlation between training speeds in swimming, cycling, and running and the actual speeds in these disciplines during the race (22). In the context of ultra-triathlons, training distance appeared to outweigh training pace in its influence on performance (175–177).

An older study highlighted the importance of training parameters such as distance, time, and experience over anthropometric factors in predicting the performance of female triathletes in short-distance races (178). Despite similar training volumes between the sexes, men tend to swim and cycle faster in training compared to women (101).

Triathletes showed a strong focus on training at low- to moderate- intensities below lactate threshold (179, 180). For example, a world-class female Olympic distance triathlete completed 74% of her swim training, 88% of her cycling training, and 85% of her running training at intensities below her individual lactate threshold (179). Also, at IRONMAN® distance, an improved performance has also been linked to extended duration of training at low to moderate intensities (180). This is also evident in other sports, including swimming (181), cycling (182), and running (183).

A recent narrative review looked at the effect of altitude training (184). The live high-train low method, meaning live at a high altitude (1,250–3,000 m) and train at low altitude or sea-level (0–1,200 m), has been used across a range of endurance-based disciplines (185). Studies have shown significant benefits for performance (186, 187).

##### 3.5.1.2 Experience

Previous participation in triathlon events (16) and achieving personal best times in both IRONMAN® and shorter distances like the Olympic distance (22, 188–190) have been robustly linked to faster IRONMAN® race times (15). Moreover, achieving a personal best time in a marathon has emerged as a strong predictor of performance in IRONMAN® races for both women (189) and men (190). In ultra-distances, the significance of race experience diminishes compared to shorter distances (172), contrary to the findings of Herbst et al. (191). Specifically, Herbst et al. found associations between the number of completed Triple Iron ultra-triathlons and personal best times in a Triple Iron ultra-triathlon with overall race time in a Deca Iron ultra-triathlon (191). However, studies across other endurance disciplines (running, cycling) have shown that the number of races completed does not correlate with overall race time (192–195).

Personal best time in an IRONMAN® triathlon is a robust predictor of race time in both women and men (22). Notably, Knechtle et al. revealed that female winners of the “IRONMAN® Hawaii” exhibit greater prior experience in IRONMAN® races and shorter distances than male winners (15). Given the association between personal best time at the IRONMAN® and race performance across sexes, it can be inferred that race experience holds greater significance than anthropometric parameters and training volume (22).

#### 3.5.2 Pacing

Pacing is a crucial element in endurance performance (196) and has been extensively studied across various triathlon distances (34, 197, 198). In IRONMAN® triathlon, pacing, alongside numerous other factors such as age, previous experience, sex, origin, anthropometry, physiology, and performance in individual disciplines, emerges as a significant predictor for race outcomes (15). Table 14 provides a summary of the main conclusions from the research on the pacing characteristics of female and male triathletes.

**Table 14:** Overview of the key findings from studies investigating the aspects of pacing in female and male triathletes.

Subjects	Distance	Observation	Outcome	Reference
*N* = 32, female = 16	Sprint distance	Differences in pacing strategy between sexes during a 5-km running test performed following a 20-km cycle	• Men run at a constant pace until lap 11 and performed the last 600 m of the 5 km race significantly faster than the initial meters. • Women performed the race with a constant pace for the entire 5 km.	(26)
*N* = 107, female = 42	Olympic distance	Relationships between athlete's pacing strategies and running performance during an international triathlon competition	• Mean running speed over the first 1,272 m of lap 1 was faster than the mean running speed over the same section during the three last laps, for both sexes. • A significant inverse correlation was observed between running speed (race) and index of running speed variability (IRSV) (race) for all triathletes. • Females demonstrated higher IRSV (race) compared to men due to greater decrease in running speed over uphill sections.	(207)
*N* = 103, female = 35	Olympic distance	Sex differences in pacing during an elite Olympic distance triathlon	• Speed over the first 222 m of the swim was associated with position and offset from the leader, at the swim finish. • Average biking speed, and both speed and pack attained in bike laps 1 and 2, influenced finishing position less in men. • Average run speed correlated better with finishing position in men than women. • Both sexes ran faster over the first 993 m than most other run sections but no clear benefit of this strategy was apparent.	(205)
*N* = 1,392, female = 129	IRONMAN® distance	Pacing strategy in the running part of 11 IRONMAN® races and cycling part of 13 IRONMAN® races in 2014	• In the IRONMAN® races evaluated, a positive pacing strategy was adopted in most races. • Women were slower than men in 6/13 cycling races, but there was no difference between women and men in the run splits. • Women used the same pacing strategy as men.	(33)
*N* = 343,345, female = 69,060	IRONMAN® distance	Effect of sex, age, and performance level on pacing of IRONMAN® triathletes	• Women spent relatively less time in swimming, running and transition time, and more time in cycling than men. • The slowest performance group was relatively faster in swimming and cycling, whereas the fastest performance group was relatively faster in running and transition time. • The younger age groups were relatively faster in swimming, running and transition time, but relatively slower in cycling.	(36)
*N* = 5,049, female = 552	Double, Triple, Quintuple, and Deca Iron ultra-triathlons	Trends of performance and sex differences in ultra-triathlons from 1985 to 2018	• Athletes spent less %time in swimming and cycling, and more %time in running as the distance of event was longer. • Women spent more %time in cycling and less %time in running in Double and Triple.	(38)
*N* = 969, female = 120	Ultra-triathlon	Trends of pacing, pacing variation, the influence of age, sex, and performance level in ultra-triathlons of different distances	• A positive pacing strategy was applied in all distances. • Faster finisher showed a significantly lower variation in cycling pacing in Double and Triple Iron ultra-triathlon and running pacing in the Triple Iron ultra-triathlon. • Pacing variations were lower in cycling than in running. • Pacing variation increased in the longer race distances. • Men paced faster than women.	(35)
*N* = 1, female = 1	Ultra-triathlon (Quintuple and Deca-Iron)	Case study: Association between pacing variation with overall performance	• The distribution of time spent in each discipline and transitions was 8.48% in swimming, 51.67% cycling, 37.91% running, and 1.94% transitions. • An even pacing strategy for both cycling and running was adopted. • The running pace has a within-day variation larger than cycling and also varies more between race days.	(240)

To mitigate premature fatigue resulting from overly aggressive cycling or running, athletes must effectively manage their metabolic energy expenditure, typically achieved through strategic adjustments of speed and intensity to optimize performance (199).

Six distinct pacing strategies have been identified: negative pacing (gradually increasing speed), positive pacing (gradual slowing), all-out pacing (maximal speed exertion), even pacing (maintaining a consistent speed), parabolic-shaped pacing (combining positive and negative pacing across race segments), and variable pacing (marked fluctuations in speed) (196). Numerous studies have shown that positive pacing is a viable strategy across various races and disciplines (200–202). Notably, elite female and male triathletes competing in the “IRONMAN® Hawaii” commonly employed positive pacing strategies during both cycling and running segments. In six out of thirteen races analyzed, female athletes notably reduced their cycling speed to a greater extent than their male counterparts, with no significant sex differences observed in speed adjustments during the running segment. (33)

In a study conducted on the “IRONMAN® Hawaii” event in 2022, it was found that male athletes displayed a predominantly negative pacing strategy during the cycling split, while female athletes maintained a more consistent pacing approach (203).

Studies have shown that men often commence the swim and cycling segments of draft-legal Olympic races with a relatively aggressive initial pace compared to women (204, 205). Despite physiological and morphological disparities between the sexes and the weight-bearing aspect of running (7, 206), both female and male triathletes typically demonstrate similar positive pacing patterns during the running segment of draft-legal Olympic-distance triathlons (204, 207).

#### 3.5.3 Nutrition

In endurance events, appropriate fueling is crucial for both safety and peak performance (208). Carbohydrates remain the primary energy source during moderate- to high intensity exercise (209), and recommendations for intake are typically based on event duration (210). However, a narrative review of Lodge et al. showed that the average carbohydrate intake in female athletes falls below current recommendations (211).

Several studies highlight that low energy availability (LEA) is a fundamental factor contributing to the Female Athlete Triad, which involves the interplay between eating disorders, irregular or absent menstrual cycles, and low bone mineral density (212–214). A study investigating low energy availability (LEA) in 30 female triathletes found that 23.3% had a monthly cycle disorder and that 20% of the participants either had, at the time of the study, or had had in the past monthly cycle disorders that could indicate an immediate LEA (215). This issue is often underestimated by both athletes and coaches, highlighting the need for greater awareness of the health-performance balance in endurance sports. Therefore, it is important to audit current guidelines for carbohydrate intake to include sport-specific recommendations and strategies for female athletes to support their health and performance (211). Furthermore, it should be noted, however, that most of the studies were not specific to triathletes, highlighting the need for triathlon-specific data on carbohydrate intake in female triathletes.

Hydration strategies also present sex-related risks, particularly with regard to exercise-associated hyponatremia (EAH) (216). EAH occurs when blood sodium levels drop below 135 mmol/L, often due to excessive fluid intake and inadequate release of vasopressin during prolonged exercise, such as an IRONMAN® race (216). This condition can lead to symptoms ranging from mild nausea and confusion to severe complications like seizures or cerebral oedema, particularly in endurance athletes. Female athletes, slower race times, extreme hot or cold external temperatures, and use of nonsteroidal anti-inflammatory drugs have been associated with a higher EAH risk (216, 217).

A study investigating EAH in female and male IRONMAN® triathletes between 1989 and 2019 confirmed that women are more likely to be hyponatremic during ultra-endurance exercise. It was found that 29.7% of women who received bloodwork testing were hyponatremic, compared to 21.2% of men. Furthermore, the women receiving bloodwork showed significantly lower serum sodium values (137.4 mEq/L vs. 139.7 mEq/L in men) (218).

#### 3.5.4 Motivation and other psychological factors

The preparation for a triathlon race requires a major commitment from the athletes. The load of trainings could influence their behavioral and psychological characteristics (219). Motivation varies between the sexes. Women's motivation often revolves around goals such as fat reduction, fitness improvement and social interaction (220–222), whereas competition and winning are more significant motivators for men. Furthermore, research by Dolan et al. suggests that most participants engage in endurance sports for reasons related to health, enjoyment, social engagement, and personal accomplishments rather than for competitive purposes (31). Interestingly, Lopez-Fernandez et al. found that female and male triathletes competing at the international level exhibit similar motivation profiles. No sex differences in sport motivation were found based on the competition level and age (223).

Table 15 summarizes the key findings of studies investigating the aspects of motivation in female and male triathletes.

**Table 15:** Overview of the key findings from studies investigating the aspects of motivation in female and male triathletes.

Subjects	Distance	Observation	Outcome	Reference
*N* = 138, female = 43	Off-road triathlon (1.5 km swim, 32 km mountain bike, 12 km trail run)	Sex differences in motivation among triathletes using the framework of self-determination theory	• Significant sex difference in amotivation, with the mean score for men higher than for women, but amotivation scores were very low. • Women and men competing at the international level in triathlon have similar motivational profiles. • Competition level and age did not influence sex differences in sport motivation.	(223)
*N* = 1,141, female = 420	No specific distance	Motivation to participate in triathlons among women and men	• Women significantly displayed more often the will to feel unity and integration, as well as the desire to gain recognition in the eyes of others, as compared to men. • For men, the desire to feel equal was significantly more important than for women. • Both women and men indicated the desire to maintain good physical condition and health, which turned out to be a significant factor.	(241)
*N* = 592, female = 200	No specific distance	Pre-race mood scores of triathletes	• Women reported higher tension scores than men, and those in the 18–25 years and 26–35 years age bands reported higher Tension scores than those in the 46–55 years age band. • Mean scores for Depression and Anger were exceptionally low and only 1.5% of triathletes, compared to the normal prevalence of ∼5%. • Mood scores did not predict triathlon performance. • Results showed an association between triathlon participation and psychological well-being.	(242)

A study investigated the psychological profile of amateur triathletes over a training period of six months prior to and after a long-distance triathlon(219). They found that the participants were more harmonious than obsessive with their triathlon's passion. Positive emotions increased until the sixth month and were significantly higher than the negative emotions (219).

Differences in psychological characteristics have been found between modalities and level of professionalism (224). Professional triathletes showed higher scores than amateur triathletes in all psychological dimensions assessed (stress control, influence of performance evaluation, motivation and mental skills). No significant differences between men and women were found. Additionally, the study showed that triathletes scored higher than cyclists in all the variables with a particularly strong effect observed in mental skills and motivation (224).

To conclude this section, future studies need to examine the factors influencing women's motivation in triathlon, including the effect of performance pressure, the presence of role models, and social influences on participation and performance. Studies on the nutrition of female triathletes are scarce. Research should therefore focus on nutrition/fluid intake and energy availability specifically for female athletes. Additionally, studies should investigate training adaptations based on the menstrual cycle to optimize performance, recovery and overall health in female triathletes. Also, the number of female participants in triathlon research should be increased and brought closer to that of men to draw safe conclusions on sex differences.

## 4 Conclusion

In conclusion, this narrative study highlights the specific problems and opportunities experienced by women in the sport of triathlon. Sex differences in participation and performance varied across distances. In female triathletes, especially in master triathletes, the participation increased and the performance improved. A significantly lower VO_2_max and a higher lactate threshold was showed in female triathletes. While the body mass was lower, the body fat percentage was higher in women. The age of peak performance in IRONMAN® triathlons is achieved between 25 and 39 years for both women and men. Personal best time in a marathon and best time in previous triathlons proved to be strong predictors of performance in IRONMAN® races.

A limitation of this narrative review is the absence of a structured assessment of study quality and potential risk of bias, particularly regarding the physiological part. Although not required for narrative reviews, such assessments are considered best practice and could have strengthened the credibility of the findings. Moreover, the lack of formal quality appraisal may limit the ability to weigh the strength of evidence across studies, especially when sample sizes are small or methodologies vary substantially. Additionally, selection bias may have occurred due to the inclusion criteria and the manual selection of studies, potentially influencing the balance of represented findings.

While there is a growing body of research on female athletes in endurance sports, most studies still focus primarily on male athletes, leading to a gap between the sexes in the currently available data. Future research should therefore aim to balance the representation of female and male athletes in the study cohorts, ensuring that the findings are relevant to both sexes. In this way, researchers can develop more comprehensive guidelines that account for the unique physiological and psychological factors influencing women in triathlon, ultimately promoting more effective and inclusive training practices.

The effect of menstruation on training and performance is an important topic that requires further research. In particular, future studies should consider longitudinal designs that track individual athletes across multiple menstrual cycles to better understand fluctuations in performance, recovery, and injury risk. Such research should also account for hormonal contraceptive use, menopausal status, and related hormonal variations.

Additionally, studies need to focus more on the impact of pregnancy and childbirth on female triathletes, as well as the influence of menopause-related hormonal changes on performance, muscle function, recovery, and risk of injury. Furthermore, the inclusion and representation of transgender athletes is another critical area given the evolving policies surrounding gender identity in competitive sports. Future studies should aim to address both the participation and performance outcomes of transgender athletes in order to contribute to a more inclusive understanding of gender in triathlon.
